# Rapid Inflammation in Mice Lacking Both SOCS1 and SOCS3 in Hematopoietic Cells

**DOI:** 10.1371/journal.pone.0162111

**Published:** 2016-09-01

**Authors:** Takashi Ushiki, Nicholas D. Huntington, Stefan P. Glaser, Hiu Kiu, Angela Georgiou, Jian-Guo Zhang, Donald Metcalf, Nicos A. Nicola, Andrew W. Roberts, Warren S. Alexander

**Affiliations:** 1 Cancer and Haematology Division, The Walter and Eliza Hall Institute of Medical Research, Parkville, Victoria, Australia; 2 Molecular Immunology Division, The Walter and Eliza Hall Institute of Medical Research, Parkville, Victoria, Australia; 3 Division of Transfusion and Regenerative Medicine, Niigata University Medical and Dental Hospital, Niigata, Japan; 4 Department of Medical Biology, The University of Melbourne, Parkville, Victoria, Australia; Emory University, UNITED STATES

## Abstract

The Suppressors of Cytokine Signalling (SOCS) proteins are negative regulators of cytokine signalling required to prevent excess cellular responses. SOCS1 and SOCS3 are essential to prevent inflammatory disease, SOCS1 by attenuating responses to IFNγ and gamma-common (γc) cytokines, and SOCS3 via regulation of G-CSF and IL-6 signalling. SOCS1 and SOCS3 show significant sequence homology and are the only SOCS proteins to possess a KIR domain. The possibility of overlapping or redundant functions was investigated in inflammatory disease via generation of mice lacking both SOCS1 and SOCS3 in hematopoietic cells. Loss of SOCS3 significantly accelerated the pathology and inflammatory disease characteristic of SOCS1 deficiency. We propose a model in which SOCS1 and SOCS3 operate independently to control specific cytokine responses and together modulate the proliferation and activation of lymphoid and myeloid cells to prevent rapid inflammatory disease.

## Introduction

Members of the suppressor of cytokine signalling (SOCS) protein family are negative feedback regulators that attenuate signal transduction initiated by specific cytokines. The SOCS family contains 8 proteins, SOCS1-7 and CIS (cytokine inducible SH2 containing protein) that are characterized by the presence of an SH2 domain that mediates interaction with signaling proteins such as the JAK kinases and/or cytokine receptors, and a C-terminal SOCS Box motif. The SOCS Box interacts with elongins B/C and cullin 5 to generate an E3 ubiquitin ligase that catalyses ubiquitination of signal transducers bound to the SOCS SH2 domain, targeting them for degradation [1,2]. In addition, SOCS1 and SOCS3 each contain an N-terminal kinase inhibitory domain (KIR), a short motif adjacent to the SH2 domain that provides a second, direct mechanism by which these proteins suppress cytokine signalling. Structural studies suggest that while SOCS3 binds JAK kinases partly via its SH2 domain, this binding surface is distinct from the canonical phosphotyrosine-binding site, leaving the latter available to bind, for example, receptor phosphotyrosines. SOCS3 inhibits JAK catalytic activity via interaction of the SOCS KIR domain with the JAK substrate-binding groove, blocking substrate access [3]. SOCS1 appears likely to act in a similar manner–the SOCS1 KIR shares high sequence similarity to that of SOCS3 and the JAK-binding surface identified in the crystal structure of SOCS3 also appears highly conserved in SOCS1 [1]. Thus, following recruitment to the signaling complex via SH2 domain interaction with phosphorylated cytokine receptors, SOCS1 and SOCS3 can suppress cytokine signaling via direct inhibition of JAK enzymatic activity as well as via SOCS Box-mediated targeting of signaling components for destruction. This model provides a biochemical basis for the specificity of SOCS actions on individual cytokine signalling pathways, which is defined by the binding affinity of specific SOCS proteins to individual JAKs and cytokine receptors [1,2].

Gene deletion studies in mice have defined essential roles of SOCS1 and SOCS3 in regulating responses to inflammatory cytokines. Mice lacking SOCS1 develop a severe inflammatory syndrome within the first two weeks after birth that is characterised by fatty degeneration of the liver, hematopoietic infiltration of multiple tissues and organs and excessive T cell activation [4,5]. This disease is caused by excessive actions of interferon-gamma (IFNγ), with mice lacking both SOCS1 and IFNγ protected from this neonatal inflammatory syndrome [6]. In *Socs1*^*-/-*^ mice, the combination of multiple pathological processes, including increased circulating IFNγ (contributed to by skewing of T lymphocyte production towards pro-inflammatory Th1 cells), deregulation of the production and function of regulatory T (Treg) cells [7,8] and the intrinsic inability of key organs such as the liver to shut down IFNγ signalling [9], conspire to drive the catastrophic disease typical of these mice.

While ablation of IFNγ prevents the neonatal disease seen in *Socs1*^*-/-*^ mice, mice lacking both SOCS1 and IFNγ nevertheless succumb in adult life to polycystic kidney disease and/or a range of inflammatory lesions [10]. *Socs1*^*-/-*^ *Ifng*^*-/-*^ mice exhibit excessive numbers of mature T cells accompanied by skewed production of CD8^+^ T cells, which express markers of activation, particularly profoundly up-regulated expression of CD44 [11], and deregulated responses to cytokines that signal via the γc receptor [12]. Indeed further studies established that a key role of SOCS1 is to control IL-15-driven activation and proliferation of naïve CD8^+^ T cells stimulated by self-ligands. In the absence of SOCS1, this deregulated proliferation results in impaired deletion of auto-reactive CD8^+^ T cells and increased potential for autoimmunity [13].

SOCS3-deficient mice fail to complete embryogenesis due to placental insufficiency driven by excess leukemia inhibitory factor (LIF) signalling [14]. Conditional inactivation of SOCS3 in blood and endothelial cells (*Socs3*^*-/*Δ*vav*^) causes inflammatory pathology in adult mice that is characterized by neutrophilia, pleural and peritoneal inflammation and multi-lineage hematopoietic infiltration of several organs. *Socs3*^*-/*Δ*vav*^ mice display deregulated responses to interleukin-6 (IL-6) and granulocyte colony-stimulating factor (G-CSF) [15,16] and IL-6 deficiency ameliorates disease and prolongs survival in these mice [17]. Interestingly, transplantation of *Socs3*^*-/*Δ*vav*^ bone marrow cells into normal recipients was insufficient to cause disease, suggesting an important contribution of SOCS3-deficient non-hematopoietic cells to inflammation [17]. Several roles for SOCS3 in T cell regulation have been reported, including influencing the balance of Th1/Th2 differentiation as well as participating in Th17 development, regulating proliferation in response to TCR ligation and affecting the production of cytokines. Actions of SOCS3 in T cells have been implicated in modulating immune and inflammatory responses, although effects appear to vary and are likely due to the control by SOCS3 of STAT3 activity, which can promote both inflammatory and anti-inflammatory responses [2,18].

SOCS1 and SOCS3 show significantly greater sequence homology to each other than to other SOCS family members, they are the only SOCS proteins to possess a KIR domain and in overexpression studies they exhibit overlapping functions for regulation of a number of cytokine pathways [19]. While the major phenotypes of mice lacking SOCS1 or SOCS3 suggest significant physiological specificity for SOCS1 (eg. IFNγ and γc cytokines) and SOCS3 (eg. G-CSF and IL-6) actions, their shared structural and biochemical characteristics imply the possibility of overlapping or redundant functions. Indeed, both SOCS1 and SOCS3 are expressed during early thymopoiesis, with expression coincident during the double negative (DN)2 and DN3 stages. While T cell precursors lacking SOCS1 exhibit a developmental block at the DN3:DN4 transition, thymocyte development in the absence of both SOCS1 and SOCS3 was more severely affected, with an earlier block in T cell differentiation at DN2 [20]. This suggests important cooperative roles for SOCS1 and SOCS3 in early T cell development and demonstrates functional overlap between these SOCS proteins. Here, we investigate the shared and overlapping roles of SOCS1 and SOCS3 in the development of inflammatory disease via the generation of mice lacking both SOCS1 and SOCS3 within the hematopoietic system. We show that loss of SOCS3, while alone causing only minor changes, significantly accelerates the pathology and inflammatory disease characteristic of SOCS1 deficiency. We propose a model in which SOCS1 and SOCS3 work independently at the cytokine and individual cell level to control the proliferation and activation of immune cells, but are both required to ensure appropriate regulation of lymphoid and myeloid cell activity in healthy immunity and to prevent excessive activity associated with rapid onset inflammatory disease.

## Materials and Methods

### Mice

IFNγ null (*Ifng*^*tm1Ts*^), SOCS1 null (*Socs1*^*-*^), SOCS3 floxed (*Socs3*^*fl*^) and Rosa26-CreERT2 mice have been described previously [5,6,21,22] and were maintained on a C57BL/6 background. In experimental mice, the Rosa26-CreERT2 allele was heterozygous. Experiments were carried out in accordance with the Australian code for the care and use of animals for scientific purposes (National Health and Medical Research Council) and specific procedures were approved by the Walter and Eliza Hall Institute of Medical Research Animal Ethics Committee (2011.031, 2014.029). Mice were housed in micro-isolators in a conventional clean facility and fed a standard irradiated diet (Ridley AgriProducts, Australia) with water *ad libitum*. To document disease onset, mice were routinely monitored at least three times per week by trained animal technicians. To minimize distress, mice were rested for a minimum of 4 weeks between bone marrow transplantation and tamoxifen treatment and at the first sign of illness (general appearance, reduced movement, ruffled fur, weight loss) were euthanized (CO_2_ asphyxiation) for analysis. Cohorts of 19–30 mice per group were monitored, allowing power to detect 2–2.5-fold increased likelihood of disease in γ*S1S3* mice relative to γ*S1* mice (p<0.05).

### Bone marrow transplantation and tamoxifen treatment

For transplantation, 8-10wk old C57BL/6 CD45.1 mice were injected with 2x10^6^ CD45.2 bone marrow cells, prepared as previously described [23], after two 5.5Gy doses of irradiation given 3h apart from a ^60^Co source (Theratron). Blood cell counts and hematopoietic chimerism were measured 5 weeks later. Tamoxifen (4.2mg for each of 2 or 3 consecutive days) was administered by oral gavage 6wk after bone marrow transplantation as previously described [24].

### Hematology and flow cytometry

Cell counts were performed on blood collected from the retroorbital plexus into Microtainer tubes containing EDTA (Sarstedt) using an Advia 2120 hematological analyser (Siemens). For total body bone marrow and blood cell counts, 1 femoral bone was calculated as 6% of whole body bone marrow [25] and a blood volume of 2mL was assumed. Flow cytometric analysis was performed on BD Bioscience LSRFortessa or LSR2 cell analysers. Cell sorting was performed on an Influx (BD Bioscience) for granulocytes or MoFlow (Beckman Coulter) for CD8^+^ T cells. Antibodies were sourced in-house: CD2 (clone RM2-5), CD45.1 (A20), CD11b (M1/70), Gr-1 (RB6-8C5), CD25 (PC61/F7); from Biolegend: CD3 (17A2), CD8 (53–6.7), PD-1 (29F.1A12), ICAM-1 (YN1/1.7.4), LPAM-1 (DATK32); eBioscience: KLRG1 (2F1); or BD Pharmingen: CD44 (IM7), CD45.2 (S450-15-2 or 104), CD49d (9C10), CD62L (MEL-14), CD69 (H1.2F3), CXCR3 (CXCR3-173), TCRβ (H57-597), LFA-1a (2D7).

### Genotyping

Southern blot analysis was performed on *Bam*HI-digested genomic DNA using a 619bp *Xba*I-*Eco*RI genomic fragment corresponding to nucleotides 117,972,193 to 117,972,872 of the *Socs3* locus on chromosome 11. The probe detected 16.6, 14 or 7.5kb fragments for wild type *Socs3* (*Socs3*^*+*^), recombined *Socs3* knockout (*Socs3*^*-*^) and un-recombined *Socs3*^*fl*^ alleles, respectively. PCR genotyping was performed using the following primers to distinguish the *Socs3*^*+*^ (613bp), *Socs3*^*fl*^ (740bp) and *Socs3*^*-*^ (288bp) alleles: 5’-ACGTCTGTGATGCTTTGCTG-3’, 5’-TCTTGTGTCTCTCCCCATCC-3’, 5’-TGACGCTCAACGTGAAGAAG-3’. PCR amplicons were separated by electrophoresis on a 2% agarose gel.

### STAT3 and STAT5 phosphorylation assays

Granulocytes (5x10^4^ CD45.2^+^CD11b^+^Gr-1^+^ cells) were stimulated with 10ng/mL recombinant human (rh) G-CSF or 10ng/mL recombinant mouse (rm) granulocyte-macrophage (GM)-CSF. The cells were fixed with PhosFlow Lyse/Fix Buffer (BD Bioscience) for 10min at 37°C, and permeabilized with Phosflow Perm Buffer III (BD Bioscience). After washing, cells were incubated for 30min with Alexa Fluor 488 anti-phospho-STAT3 (pY705, BD Phosflow) or with Alexa Fluor 488 anti-phospho-STAT5 (pY694, BD Phosflow), washed and analysed using a Fortessa flow cytometer (BD Bioscience).

### Hematopoietic colony forming assays

2.5x10^4^ bone marrow cells were resuspended in 0.3% agar in modified Dulbecco’s medium (DME) with 20% FCS and stimulated with rhG-CSF (10^3^U/mL, Neupogen, Amgen), rh erythropoietin (EPO, 2U/mL, Eprex 4000, Janssen-Cilag Pty Ltd), rmGM-CSF (10ng/mL), rmM-CSF (10ng/mL), rmIL-6 (100ng/mL), rmIL-3 (10ng/mL), rm stem cell factor (SCF, 100ng/mL, in house), or combinations thereof. Cells were cultured for 7d at 37°C in a humidified atmosphere of 10% CO_2_ in air. Cultures were fixed, dried onto glass slides, and stained for acetylcholinesterase, Luxol fast blue, and haematoxylin.

### Culture and stimulation of CD8^+^ T cells

CD8^+^ cells were enriched from spleen cell preparations via positive selection using anti-CD8a (Ly-2) microbeads (Miltenyi Biotech) and then CD45.2^+^CD3^+^CD8^+^ T cells were sorted by flow cytometry. 5x10^4^ cells were seeded in wells of a 96-well plate with 100μL RPMI 1640 medium supplemented with 1mM sodium pyruvate, 10mM HEPES, 50μM mercaptoethanol, and 10% FCS with 40ng/mL rmIL-15 (Peprotech). Alternatively, wells were coated with anti-CD3 (5μg/mL, clone 145-2C11) and anti-CD28 antibodies (1μg/mL, clone 37.51) at 4°C overnight and then cells were added with 20ng/mL rmIL-2 (R&D systems) for 2 or 3d.

### Cytokine Bio-Plex assay

Concentrations of 23 cytokines (IL-1α, IL-1β, IL-2, IL-3, IL-4, IL-5, IL-6, IL-9, IL-10, IL-12p40, IL-12p70, IL-13, IL-17, Eotaxin, G-CSF, GM-CSF, IFNγ, KC, MCP-1, MIP1α, MIP1β, RANTES, TNFα) were determined in serum or supernatant from either IL-15 stimulated or IL-2/anti-CD3/28 stimulated CD8^+^ T cells, by Bio-Plex (Bio-Rad laboratories). Data were analysed using the Bio-Plex Manager 6.0 software.

### Macrophage culture

2x10^7^ bone marrow cells were cultured in DME supplemented with 20% L-cell-conditioned medium and 10% FCS in 10cm tissue culture-treated dishes on day 1. Non-adherent cells were split into two non culture-treated plates on day 2. Additional 10% L-cell-conditioned medium was added on days 3 and 5. Macrophages were harvested with cell dissociation buffer (Gibco) and 1x10^6^ cells were seeded in well of a 6 well plate on day 6. On day 7, cells were washed, starved for 3h in DME containing 0.1% BSA then pulsed with rmIL-6 (100ng/mL for 30min). Cells were washed, lysed in KALB buffer and the extract fractionated by SDS-PAGE for western blot analysis using antibodies to SOCS3 (IBL 18391), STAT1 (BD 610116 and BD 610186), phosphorylated STAT1 (CST 9171), STAT3 (Santa Cruz sc-482), phosphorylated STAT3 (CST 9145), STAT5 (Zymed 13–3600), and phosphorylated STAT5 (Upstate 05–495) as described [26].

### Histopathology

Organs were fixed in 10% formalin, and embedded in paraffin for sectioning and staining with haematoxylin and eosin. Photomicrographs were taken using a Nikon Eclipse E600 microscope and Zeiss Axiocam MRc5 camera.

### Statistics

Unless otherwise stated, data were analysed using analysis of variance (ANOVA) corrected for multiple testing. P-values for specific comparisons were determined using GraphPad Prism. Other comparisons analysed are indicated in the Figure legends.

## Results

### Generation of mice lacking SOCS1 and SOCS3 in hematopoietic cells

Analysis of functional redundancy between SOCS1 and SOCS3 *in vivo* has been complicated by the embryonic or early post-natal lethality of mice lacking these proteins due to the uncontrolled actions of IFNγ and LIF respectively. Therefore, to explore the combined loss of SOCS1 and SOCS3 in inflammation, we determined the effects of post-natal inactivation of SOCS3 on disease development and severity in the *Ifng*^*-/-*^*;Socs1*^*-/-*^ model of adult-onset inflammatory disease. To overcome the embryonic lethality of SOCS3-deficiency we used a tamoxifen-inducible cre-recombinase, *Rosa26-CreERT2* (*ERT2*) [22] in combination with a conditional *Socs3* allele (*Socs3*^*fl*^ [27]). To focus on SOCS actions in hematopoietic cells, highly reconstituted (>80%) wild-type recipients of bone marrow from specific donor genotypes on an IFNγ-deficient background were generated and then either treated with tamoxifen or vehicle to generate the test and control hematopoietic genotypes: solely IFNγ-deficient (γ, vehicle-treated recipients of *Ifng*^*-/-*^*;Socs1*^*+/+*^*;Socs3*^*fl/fl*^*;ERT2* marrow); SOCS1-deficient (γ*S1*, vehicle-treated recipients of *Ifng*^*-/-*^*;Socs1*^*-/-*^*;Socs3*^*fl/fl*^*;ERT2* marrow); SOCS3-deficient (γ*S3*, tamoxifen-treated recipients of *Ifng*^*-/-*^*;Socs1*^*+/+*^*;Socs3*^*fl/fl*^*;ERT2* marrow); and SOCS1, SOCS3 double deficient (γ*S1S3*, tamoxifen-treated recipients of *Ifng*^*-/-*^*;Socs1*^*-/-*^*;Socs3*^*fl/fl*^*;ERT2* marrow) (S1 Fig). Mice with hematopoiesis lacking only SOCS3 on a *Ifng*^*+/+*^ background (*S3*, tamoxifen-treated recipients of *Ifng*^*+/+*^*;Socs1*^*+/+*^*;Socs3*^*fl/fl*^*;ERT2* marrow) were included as a control, as were several controls of mice with functionally normal hematopoiesis (*S3*^*fl*^, vehicle-treated recipients of *Ifng*^*+/+*^*;Socs1*^*+/+*^*;Socs3*^*fl/fl*^*;ERT2* marrow); and either tamoxifen treated (TAM+) or vehicle treated (TAM-) recipients of *Ifng*^*+/+*^*;Socs1*^*+/+*^*;Socs3*^*+/+*^*;ERT2* marrow (S1 Fig). Near-complete tamoxifen-induced inactivation of the *Socs3* allele was confirmed in the hematopoietic organs of *Socs3*^*fl/fl*^*;CreERT2* bone marrow (S1 Fig) and was confirmed in the spleens of selected experimental animals at the time of analysis. The studies described below refer to analyses performed at indicated times following treatment with either tamoxifen or vehicle 6 weeks after transplantation (S1 Fig).

### Combined hematopoietic loss of SOCS1 and SOCS3 causes rapid inflammatory disease

**Fig 1:**
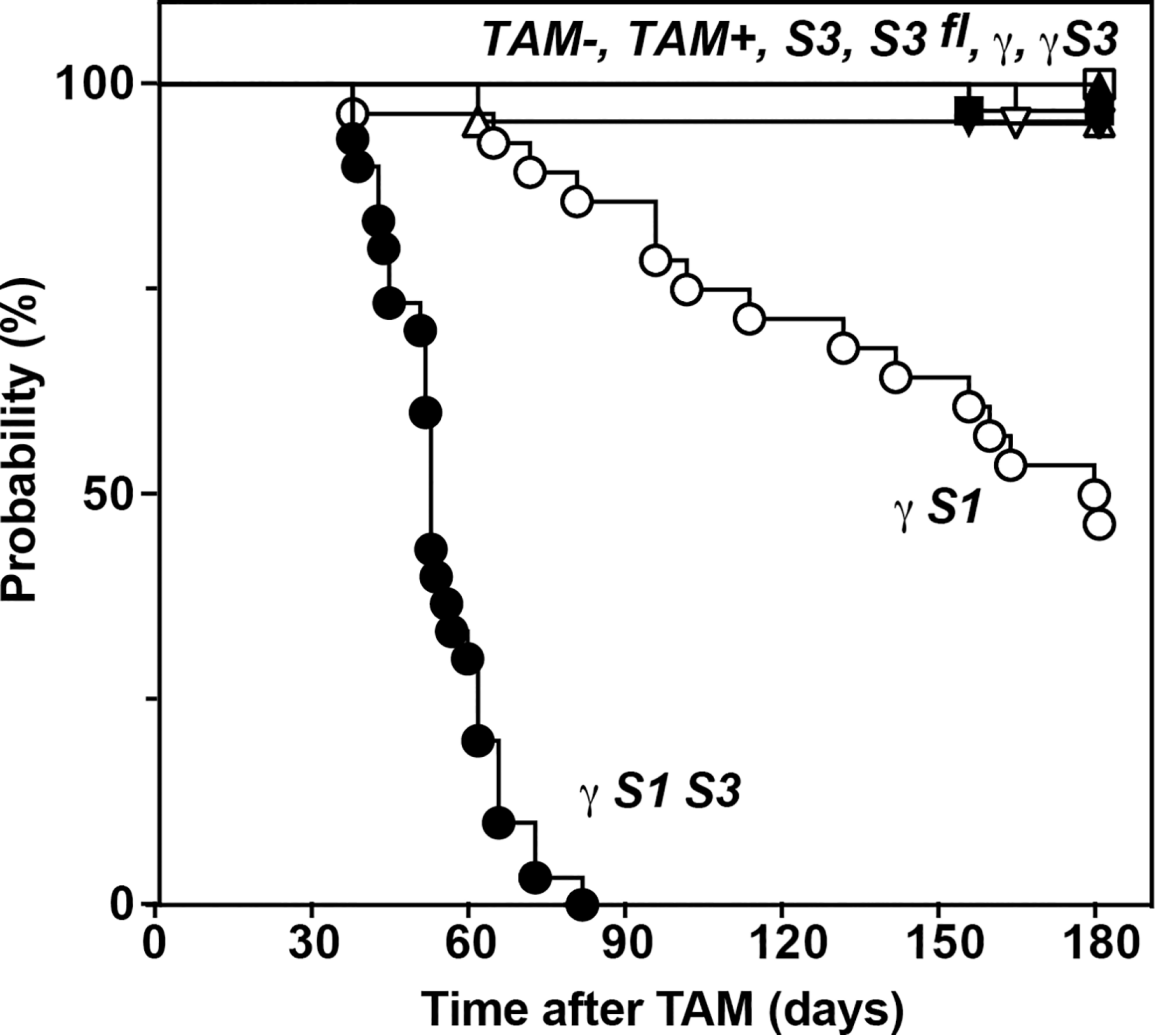
Rapid disease onset in mice lacking SOCS1 and SOCS3 in hematopoietic cells. Onset of disease in mice with hematopoietic cells lacking SOCS1 (γ*S1*, ○) or both SOCS1 and SOCS3 (γ*S1S3*, ●) compared with γ (▼),γ*S3* (▼), *S3* (■), *S3*^*fl/fl*^ (□) or control *TAM+* (▲) or *TAM-* (△) mice. Mice were generated as described in S1 Fig. p<0.0005 for pairwise comparison of survival of γ*S1* with *TAM-* or γ and for γ*S1S3* with γ*S1* or γ*S3*, Mantel-Cox Log-rank test, n = 19–30 mice per group.

As expected, recipients of control bone marrow (*S3*^*fl*^, *TAM+* or *TAM-*) showed no significant disease over a 6-month observation period. Similarly, *S3*, γ and γ*S3* mice remained healthy (Fig 1). In contrast, and consistent with previous studies of unmanipulated *Ifng*^*-/-*^*;Socs1*^*-/-*^ mice [10], mice with SOCS1-deficient hematopoiesis became unwell with half the cohort developing disease within 180 days post treatment (γ*S1*, Fig 1). A more rapid course of disease was evident in mice with blood cells lacking both SOCS1 and SOCS3: all mice became unwell with a mean survival of only 52 days following tamoxifen-induced deletion of SOCS3 (γ*S1S3*, Fig 1). Mice were analysed upon initial signs of disease (referred to hereafter as moribund or diseased). To allow time-matched comparisons, data from diseased γ*S1S3* mice (mean onset, 52 days) were commonly compared with that of mice of other genotypes at day 50 while data from diseased γ*S1* mice (mean survival time, 180 days) were commonly compared with that of mice of other genotypes at day 180.

**Fig 2:**
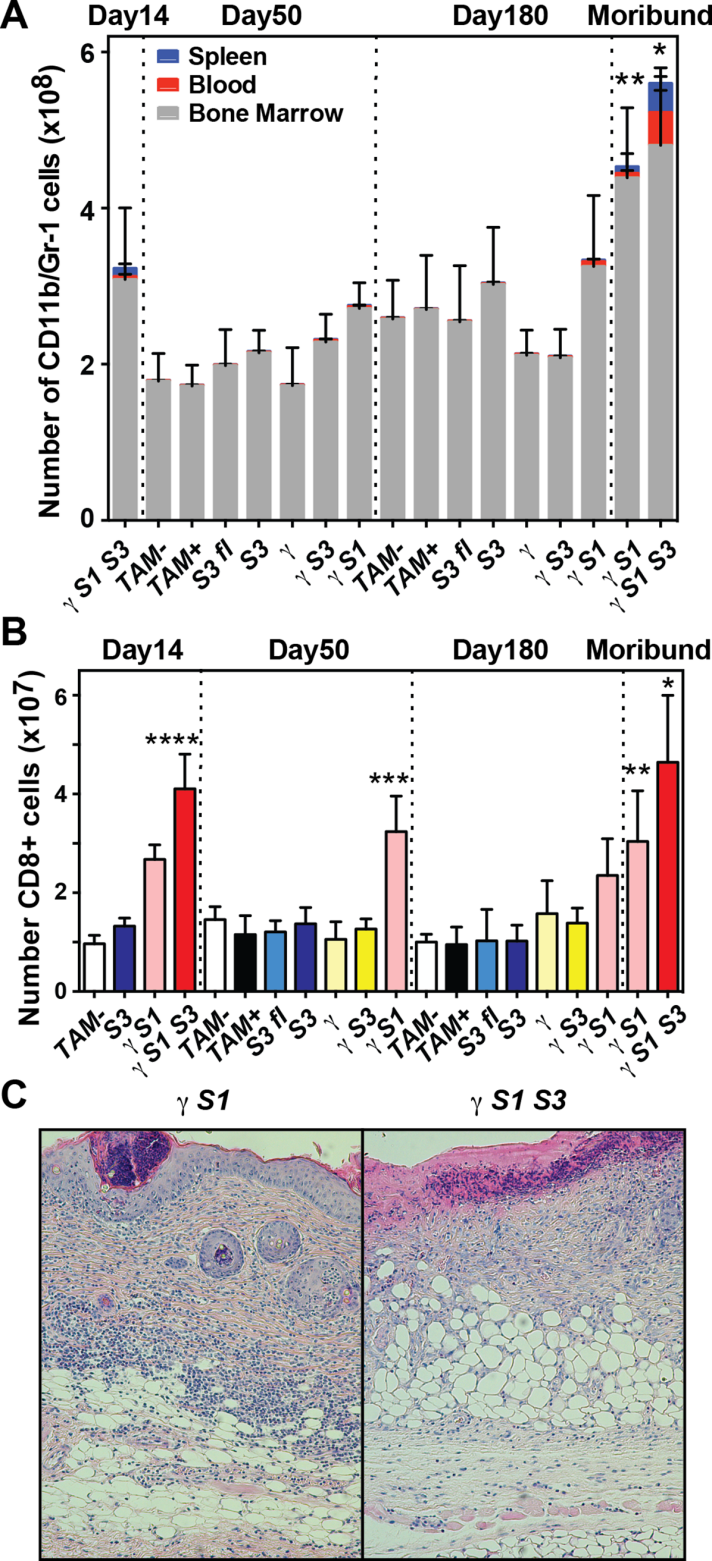
Altered bone marrow granulocyte and spleen T cell numbers and pathology in mice lacking SOCS1 and/or SOCS3. (**A**) Numbers of neutrophils in the spleen, bone marrow and blood of mice at 14, 50 and 180d following tamoxifen or vehicle treatment, or upon signs of disease (moribund). Mean ± SD is shown with * p<0.05 for comparison of γ*S1S3* (moribund) with all genotypes at day 50 for each of spleen, blood and bone marrow, with γ*S1* (moribund, for spleen and blood only) and with γ*S1S3* (day 14, for each of spleen, blood and bone marrow). ** p<0.05 for comparison of γ*S1* (moribund) with all genotypes (for bone marrow only) at day 180 including γ*S1* (non-moribund, day 180), one-way ANOVA with Tukey’s multiple comparisons test, n = 7–20 mice per group. (**B**) Number of CD8^+^ T cells in the spleens of mice at 14, 50 and 180d following tamoxifen or vehicle treatment, or when moribund. Mean ± SD is shown with * p<0.05 for comparison of γ*S1S3* (moribund) with all genotypes at day 50 and with γ*S1* (moribund). **** p<0.05 for comparison of γ*S1S3* (day 14) with all genotypes at day 14. ** p<0.05 for comparison of γ*S1* (moribund) with all genotypes at day 180 except γS1 (non-moribund, day 180). *** p<0.05 for comparison of γ*S1* (day 50) with all other genotypes at that time. One-way ANOVA with Tukey’s multiple comparisons test, n = 5–15 mice per group. (**C**) Photomicrographs showing inflammation and mixed hematopoietic infiltration of the skin in moribund mice with bone marrow cells lacking SOCS1 (γ*S1*) or both SOCS1 and SOCS3 (γ*S1S3*).

Moribund γ*S1S3* mice often displayed inflammatory skin lesions and/or ulcers and upon autopsy exhibited splenomegaly and thymic atrophy. In the blood, there was a striking elevation in the numbers of white blood cells, attributable primarily to a marked neutrophilia (Fig 2A; S2 Fig; S1 Table). Excess neutrophils were also evident in the bone marrow and spleen (Fig 2A; S2 Fig; S3 Fig). Moribund γ*S1S3* mice also displayed a marked expansion of T cells in the spleen, significantly skewing the B cell:T cell ratio (S3 Fig), and that was attributable to a selective expansion of CD8^+^ T cells (Fig 2B, S2 Fig; S3 Fig). The CD8^+^ T cell excess and splenomegaly were already evident in γ*S1S3* mice 14 days after induction of SOCS3 deletion, but were exacerbated in moribund mice (Fig 2B; S2 Fig). Histological examination of moribund γ*S1S3* mice revealed mixed infiltration (neutrophils, monocytes, eosinophils and lymphocytes) at numerous sites of inflammation including the skin, liver, lung and duodenum (Fig 2C; S4 Fig).

Moribund γ*S1* mice shared many of these clinical and pathological features, including splenomegaly, thymic atrophy and the accumulation of neutrophils in the bone marrow and CD8^+^ T cells in the spleen (Fig 2A and 2B; S2 Fig). The excess neutrophils in the bone marrow of γ*S1* and γ*S1S3* mice was progressive, being significantly greater in moribund mice compared with time-matched controls, and this was also true of neutrophil numbers in the spleen and blood of γ*S1S3* mice (Fig 2A; S2 Fig). In contrast, the accumulation of excess CD8^+^ T cells was evident at earlier time points in both γ*S1* and γ*S1S3* mice (Fig 2B; S2 Fig).

In mice lacking SOCS3 in hematopoietic cells, either in the presence (*S3*) or absence (γ*S3*) of IFNγ and at all times examined, spleen weight, neutrophil numbers in the blood, spleen and bone marrow, as well as spleen CD8^+^ T cell numbers and the ratios of B220^+^/CD3^+^ and CD4^+^/CD8^+^ cells, were no different to those in mice with functionally normal hematopoiesis (*S3*^*fl*^, *TAM+* or *TAM-*, Fig 2A and 2B; S2 Fig; S3 Fig). Accordingly, in some subsequent experiments SOCS3-deficient cells were sourced from either genotype. Typically, *TAM-* cells were used as functionally normal controls.

Together, these data establish that when restricted to the hematopoietic system, loss of SOCS3, while alone causing minor changes, significantly accelerates the pathology and inflammatory disease characteristic of SOCS1 deficiency.

### Loss of SOCS3 abrogates the need for excessive G-CSF production to drive SOCS1-deficient inflammatory disease

**Fig 3:**
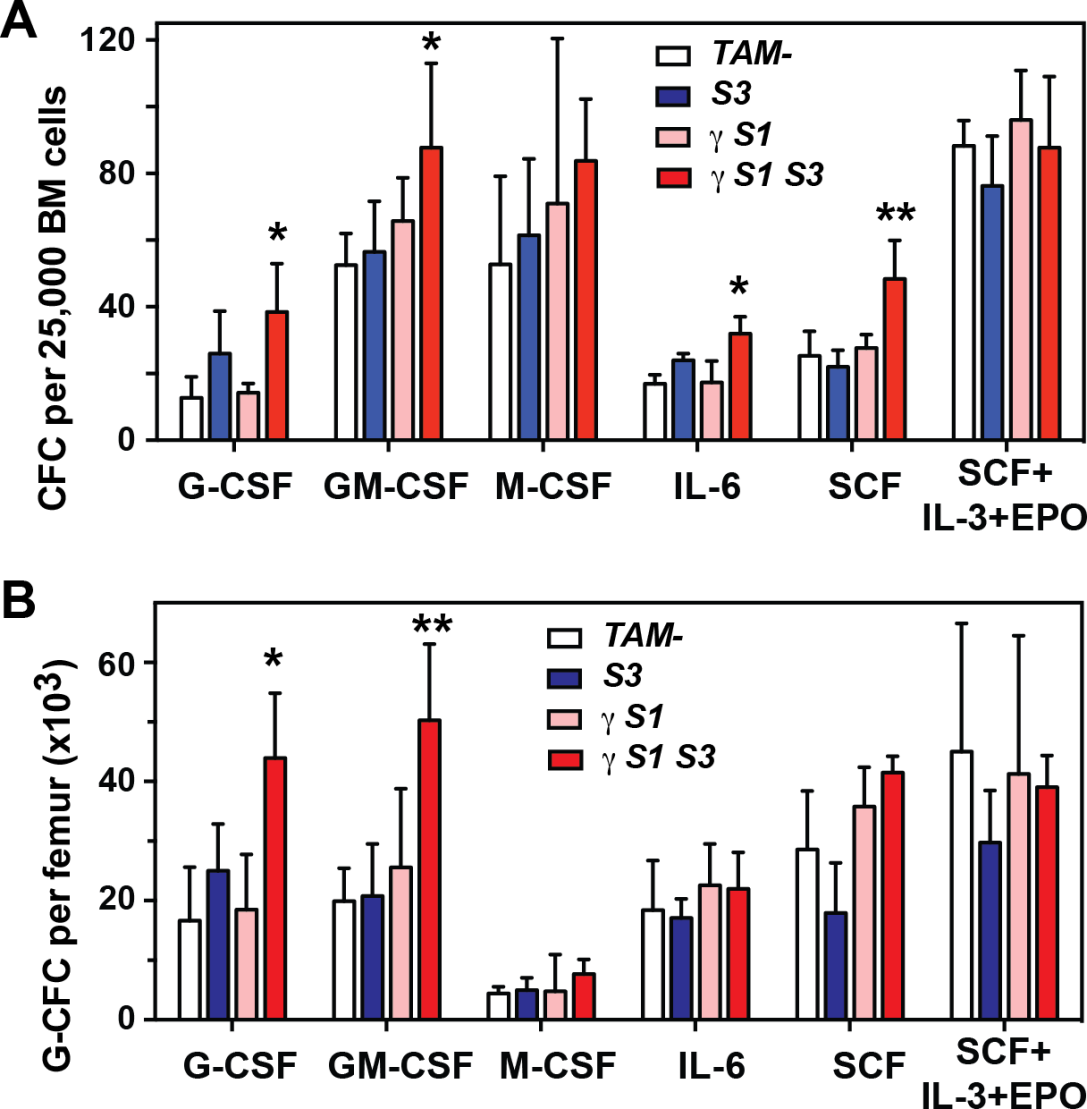
Hematopoietic colony formation by bone marrow cells from mice lacking SOCS proteins. Total number (**A**) or number of granulocyte (**B**) colonies in agar cultures of bone marrow cells from mice 14d after tamoxifen or vehicle treatment. Cultures were stimulated with G-CSF, GM-CSF, M-CSF, IL-6, SCF or the combination of SCF, IL-3 and EPO. Mean ± SD is shown with * p<0.05 for comparison of γ*S1S3* with TAM- and γ*S1* but not *S3*, ** p<0.05 for comparison of γ*S1S3* with all other genotypes, one-way ANOVA with Tukey’s multiple comparisons test, n = 3–4 mice per group.

To investigate the contribution of myeloid cytokine regulation to the exacerbated neutrophilia in γ*S1S3* mice, the numbers of cytokine-responsive colony forming cells in the bone marrow were enumerated at day14 after tamoxifen treatment. As previously observed in bone marrow cells lacking SOCS3 [16], an increased proportion of colony-forming cells (CFC) was evident in γ*S1S3* marrow cells stimulated with G-CSF or IL-6, as were total numbers of G-CSF-responsive femoral granulocyte-CFC, however these were not significantly greater in magnitude than that observed in marrow lacking SOCS3 alone (*S3*; Fig 3A and 3B). Significantly however, the total number per femur of granulocyte colony-forming cells was selectively elevated in cultures of GM-CSF-stimulated γ*S1S3* bone marrow; this was not observed in *S3*, γ*S1* or functionally normal cell cultures (Fig 3B).

**Fig 4:**
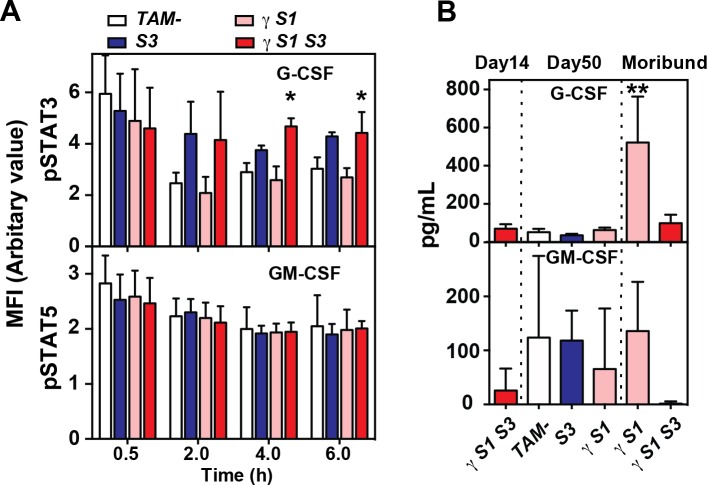
Cytokine signalling and serum concentrations in mice lacking SOCS proteins. (**A**) PhosFlow measurement of phosphorylated STAT3 (upper panel) and STAT5 (lower panel) in granulocytes prepared from mice 14d after tamoxifen or vehicle treatment. Cells were stimulated with G-CSF or GM-CSF for the times indicated. MFI, mean fluorescence intensity. Mean ± SD is shown with * p<0.05 for comparison of γ*S1S3* with *TAM-* and γ*S1* but not *S3*, one-way ANOVA with Tukey’s multiple comparisons test, n = 3 samples per time point. (**B**) G-CSF and GM-CSF in the serum of mice at the indicated times after tamoxifen or vehicle treatment. Mean ± SD is shown with ** p<0.05 for comparison of γ*S1* (moribund) with all other genotypes and times, one-way ANOVA with Tukey’s multiple comparisons test, n = 6–8 mice per group.

**Fig 5:**
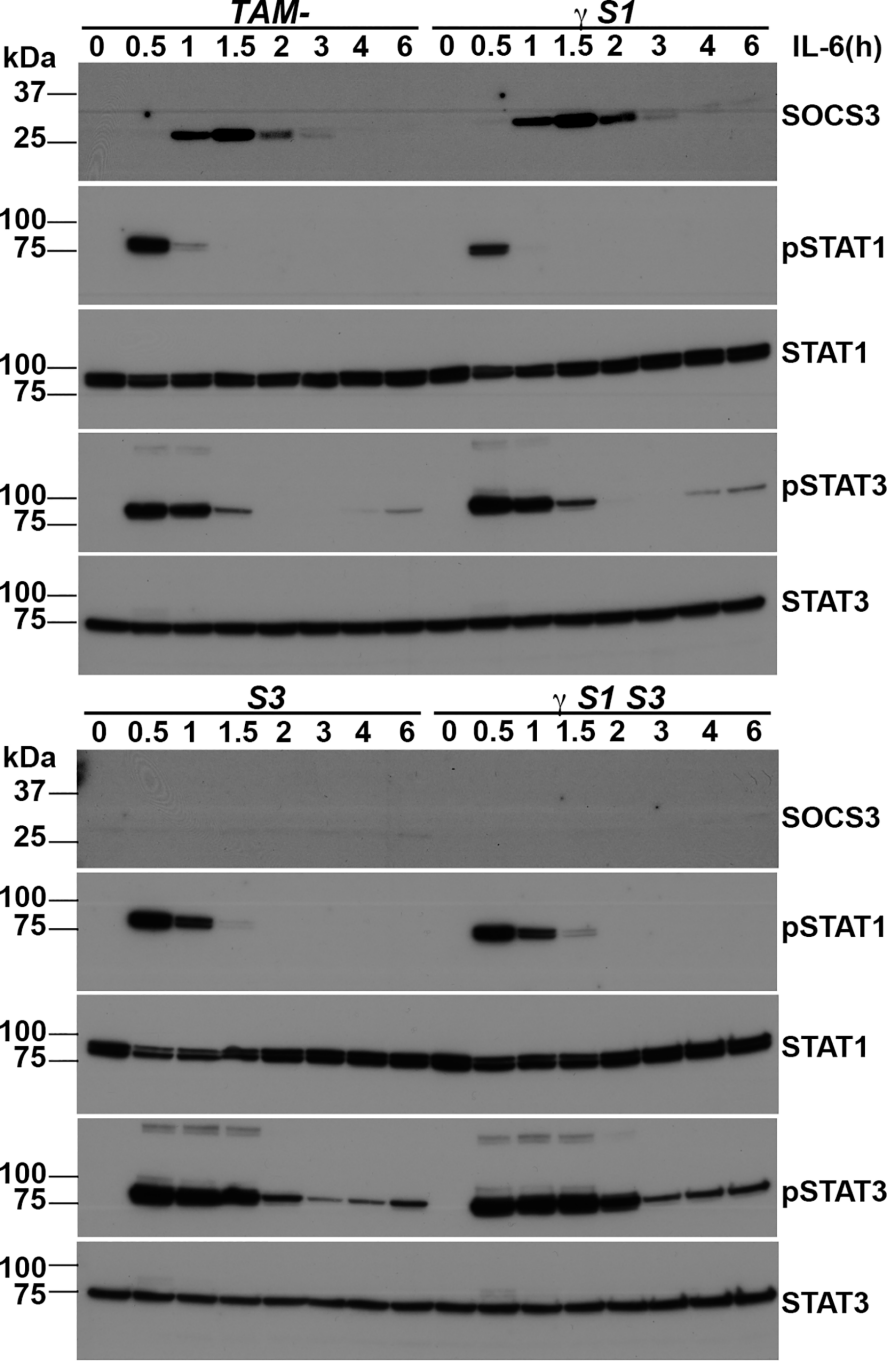
Interleukin-6 signalling in mice lacking SOCS proteins. Western blot analysis of macrophages prepared from mice 14d after tamoxifen or vehicle treatment. The cells were stimulated with IL-6 and lysates were prepared at the times indicated. Proteins were separated by polyacrylamide gel electrophoresis, transferred to membranes and probed with antibodies to the molecules indicated at the right. Replicate filters were prepared from the same lysates and probed with pSTAT1 and pSTAT3 and subsequently with STAT1 and STAT3 and SOCS3. Images were scanned and cropped to prepare the Figure; for uncropped images see S6 Fig.

In the absence of SOCS3, prolonged responses to G-CSF accompany the accumulation of neutrophils and the development of inflammation [16]. Prolonged phosphorylation of STAT3 was evident in G-CSF-stimulated granulocytes from γ*S1S3* mice, but this did not differ significantly in magnitude from that in the sole absence of SOCS3 (Fig 4A; S5 Fig). The kinetics of STAT5 phosphorylation in response to GM-CSF was normal in granulocytes purified from each of *S3*, γ*S1*, and γ*S1S3* mice (Fig 4A; S5 Fig). Similarly, in bone marrow-derived macrophages established from recipient mice at 14 days after treatment, IL-6 stimulation resulted in prolonged phosphorylation of STAT3 in the absence of SOCS3 but not SOCS1, as previously reported [15] and this was not further altered in cells lacking both SOCS1 and SOCS3 (Fig 5 and S6 Fig).

Although inflammatory disease was accelerated and exacerbated in γ*S1S3* mice relative to that observed in γ*S1* mice, G-CSF was only present in elevated concentrations in the serum of moribund γ*S1* mice and not γ*S1S3* animals (Fig 4B). While GM-CSF was detected in the serum of all mice, it was not present at elevated levels in moribund γ*S1* or γ*S1S3* mice (Fig 4B).

Together these data suggest that while neutrophilic inflammation contributes to the disease afflicting both γ*S1* and γ*S1S3* mice, responses to cytokines such as G-CSF and IL-6, known to be deregulated in the absence of SOCS3, appear no further intrinsically deregulated in cells lacking both SOCS1 and SOCS3. However, reflecting the key role for SOCS3 in regulating responses to granulopoietic cytokines, the accelerated onset of neutrophilia and inflammatory disease in γ*S1S3* mice appears driven by hyper-responsiveness to normal levels of G-CSF (driven by loss of SOCS3), while the later onset neutophilia and disease in γ*S1* mice is dependent upon the development of high levels of G-CSF.

### Loss of SOCS1 increases the number of CD44^hi^ CD8^+^ cells

**Fig 6:**
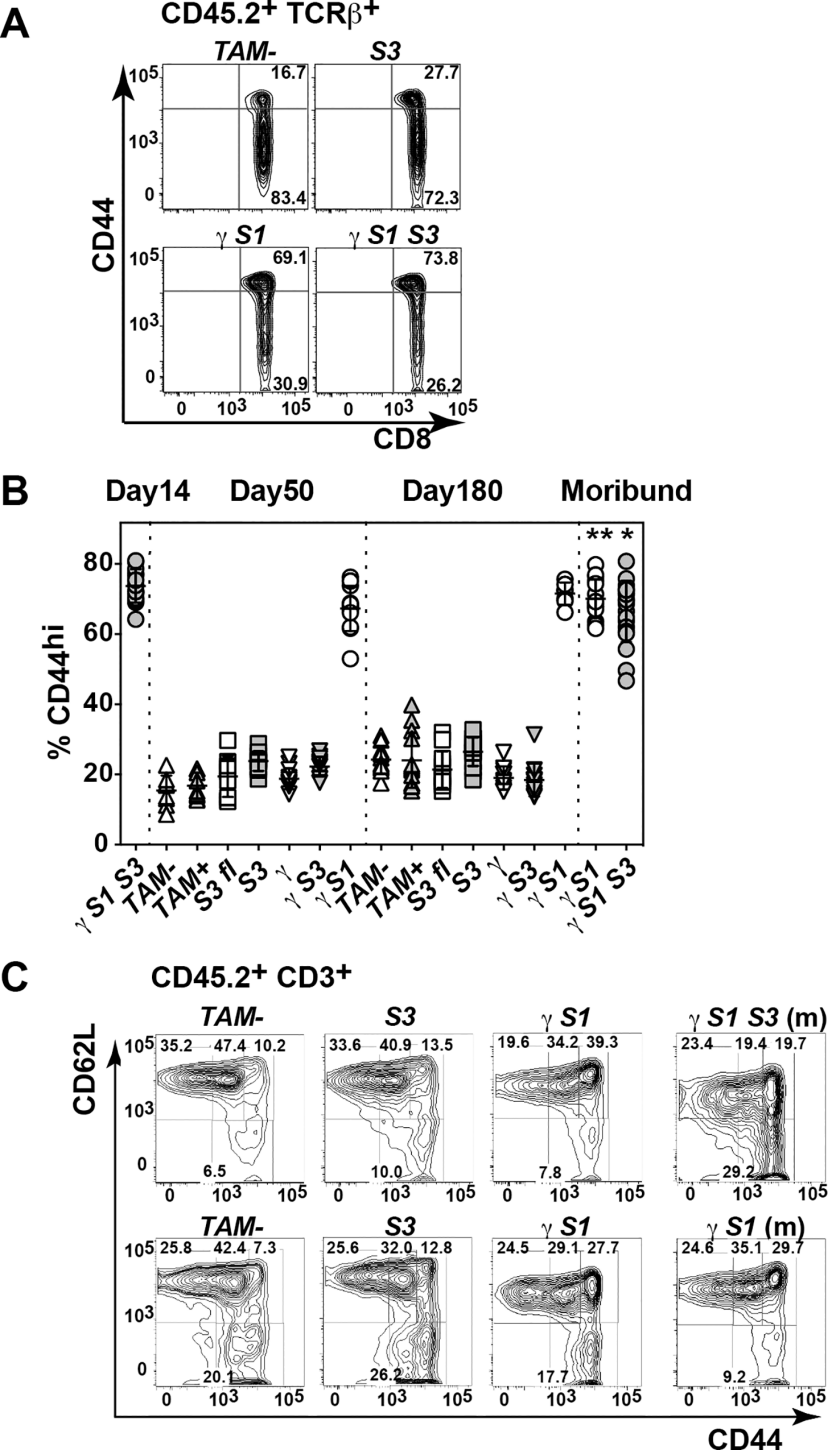
CD44 expression on T cells in mice lacking SOCS proteins. (**A**) Representative flow cytometry profiles of CD8 and CD44 expression on TCRβ^+^ T cells in spleens mice 14d after tamoxifen or vehicle treatment. (**B**) Proportions of CD8^+^ T cells expressing high levels of CD44 (CD44^hi^) in spleens of mice at the times indicated after tamoxifen or vehicle treatment. (**C**) Proportions of naïve (CD44^-^ CD62L^+^), central memory (CD44^+^ CD62L^+^) and effector memory (CD44^+^ CD62L^-^) CD3^+^ T cells in spleens of mice 50d (top panels) or 180d (bottom) after tamoxifen or vehicle treatment (m, moribund). Mean ± SD is shown with * p<0.05 for comparison of γ*S1S3* (moribund) with all genotypes at day 50 except γ*S1* (non-moribund day 50). ** p<0.05 for comparison of γ*S1* (moribund) with all genotypes at day 180 except γ*S1* (non-moribund, day 180), one-way ANOVA with Tukey’s multiple comparisons test, n = 7–20 mice per group.

The CD8^+^ lymphocytosis that characterised the inflammatory disease in γ*S1* and γ*S1S3* mice was an early phenotype, evident within 50 days of treatment and was specific to SOCS1-deficient marrow recipients: γ*S1* and γ*S1S3* but not *S3*^*fl*^, *S3*, γ*S3*, γ nor *TAM+*/- mice (Fig 2B; S2 Fig). Within this expanded CD8^+^ population, CD8^+^CD44^hi^ cells were selectively and significantly increased (Fig 6A and 6B). In moribund γ*S1S3* mice, the majority of CD3^+^CD44^hi^ cells expressed low levels of CD62L, suggestive of accumulation of effector memory T cells. This was not observed in moribund γ*S1* mice (Fig 6C).

**Fig 7:**
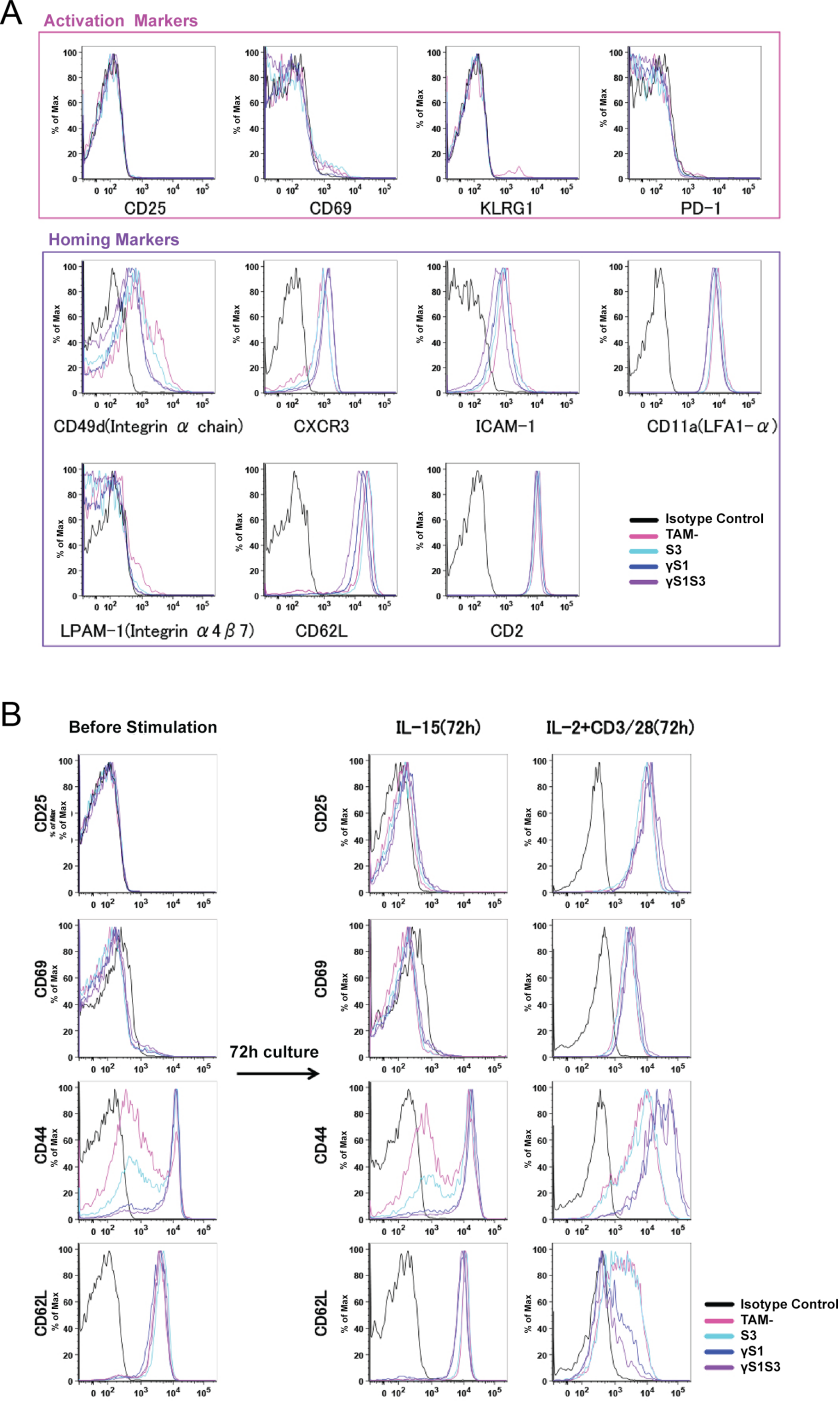
Expression of activation and homing markers on T cells from mice lacking SOCS proteins. (**A**) Representative flow cytometry profiles showing expression of activation and homing markers as indicated on CD8^+^ CD44^high^ cells from spleens of mice 14d after tamoxifen or vehicle treatment. (**B**) CD8^+^ T cells were purified from spleens of mice 14d following tamoxifen or vehicle treatment. The cells were stimulated for 72 hours in culture in the presence of IL-15 or IL-2 plus anti-CD3/CD28. Expression of CD25, CD69, CD44 and CD62L before and after culture in a representative experiment is shown.

**Fig 8:**
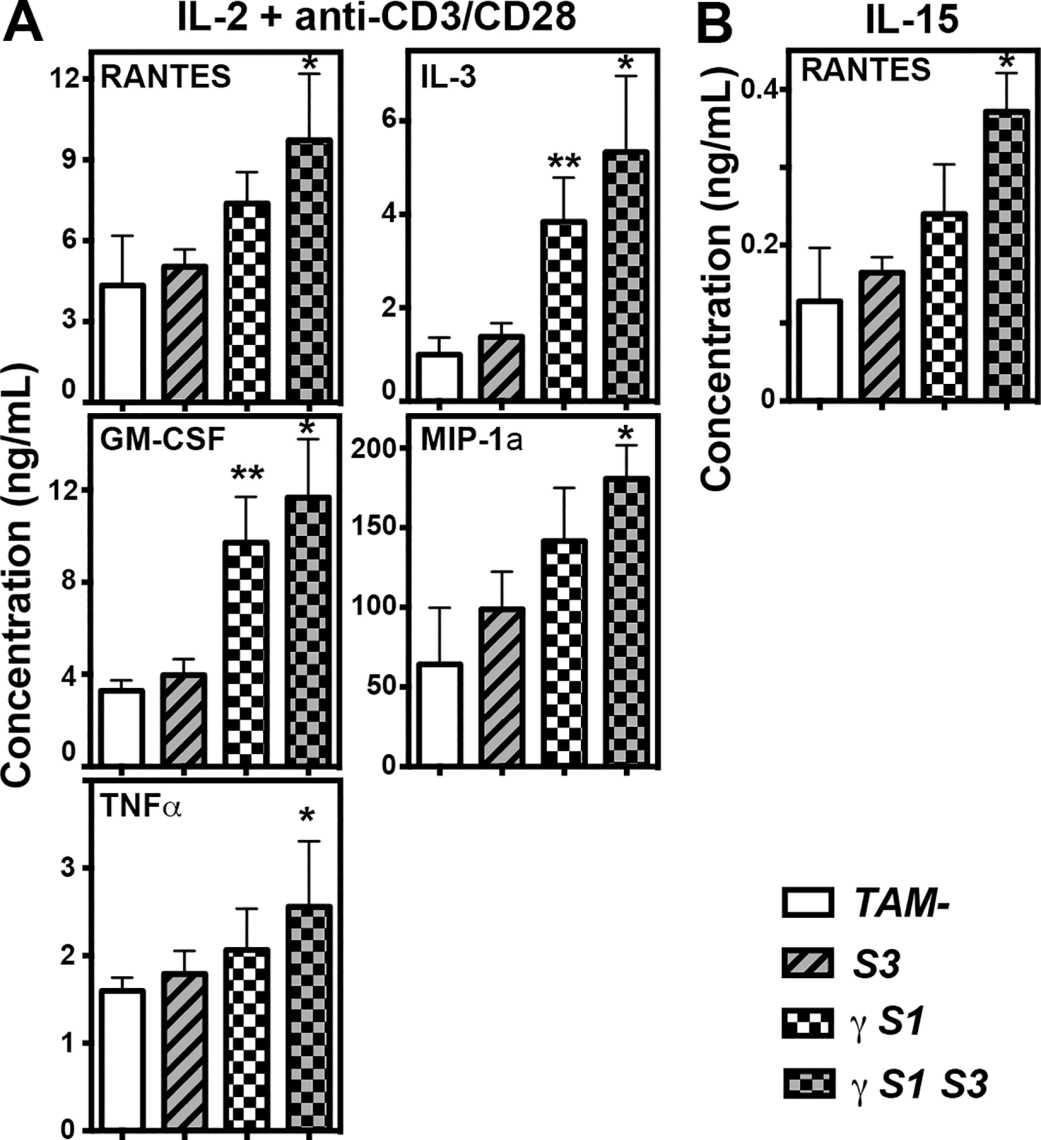
Cytokine and chemokine production by T cells from mice lacking SOCS proteins. Concentrations of cytokines/chemokines in supernatant of CD8^+^ T cells 48hr after stimulation with IL-2 plus anti-CD3/CD28 (**A**) or IL-15 (**B**). Mean ± SD is shown. CD45.2^+^CD3^+^CD8^+^ T cells were flow-sorted from spleens of mice 14d after tamoxifen or vehicle treatment. Mean ± SD is shown with * p<0.05 for comparison of γ*S1S3* with TAM- and *S3*. ** p<0.05 for comparison of γ*S1* with TAM- and *S3*, one-way ANOVA with Tukey’s multiple comparisons test, n = 6–7 mice per group.

The phenotype of the CD8^+^CD44^hi^ T cell population was further examined at 14 days following treatment, when this population had already accumulated in γ*S1* and γ*S1S3* mice but prior to development of illness. In γ*S1* and γ*S1S3* mice, as well as *S3 KO* and *TAM-* controls, these CD8^+^CD44^hi^ cells were uniformly negative for expression of activation markers, including CD25, CD69, KLRG-1 and PD-1 (Fig 7A). However, these cells expressed a number of homing markers including CD49d, CXCR3, ICAM-1 and CD11a (Fig 7A). CD8^+^ T cells were purified from the spleens of mice 14 days following treatment and stimulated in culture for 72 hours in the presence of IL-15 or IL-2 plus CD3/CD28. As expected, the latter combination stimulus caused cells of all genotypes to up-regulate expression of CD25 and CD69. However, while γ*S1* and γ*S1S3* T cells were already CD44^hi^, as described above, they further up-regulated CD44 and expressed significantly higher levels than those induced by IL-2 plus CD3/CD28 on control T cells or cells lacking only SOCS3 (Fig 7B). Similarly, unlike control and SOCS3-deficient T cells, γ*S1* and γ*S1S3* cells uniformly down-regulated CD62L and to a greater extent than observed in the control cultures (Fig 7B). Stimulation of T cells with IL-15 did not result in significant changes in expression of CD25, CD69, CD44 or CD62L, although some modest down regulation of the latter marker was evident specifically in γ*S1* and γ*S1S3* cells (Fig 7B). Examination of cytokine and chemokine production by T cells stimulated with IL-2 plus CD3/CD28 revealed significantly higher production of pro-inflammatory and/or chemotactic agents RANTES, MIP1α, IL-3, GM-CSF and TNFα by γ*S1S3* cells compared with *TAM-* and *S3* cells, that was often also observed from γ*S1* cells. Increased production of RANTES was also evident in IL-15-stimulated γ*S1* and γ*S1S3* cultures (Fig 8). Notably, production of anti-inflammatory cytokines, including IL-10, was similar in IL-2 plus CD3/CD28 stimulated cultures of T cells of all genotypes.

## Discussion

Coordinated regulation of the immune system by SOCS1 and SOCS3 was investigated by generation of mice lacking both these proteins in adult hematopoiesis. The focus of this study was to investigate whether loss of SOCS3 influenced the development of autoimmune pathology in mice lacking SOCS1 and IFNγ. The embryonic lethality of SOCS3-deficiency was overcome by using a conditional *Socs3* allele inactivated via a tamoxifen-inducible cre-recombinase. The specific actions of SOCS proteins in hematopoietic cells were investigated by analysis of highly reconstituted wild-type recipients of bone marrow from specific donor genotypes.

Recipients of bone marrow lacking SOCS1 and IFNγ (γ*S1* mice) developed a delayed-onset inflammatory disease with features typical of those previously described in unmanipulated *Socs1*^*-/-*^ *Ifng*^*-/-*^ mice [10] including multi-lineage hematopoietic infiltration of the major organs and accumulation of neutrophils. Excessive numbers of CD8^+^ CD44^hi^ T cells, a defining feature of the absence of SOCS1 [11,13] were evident in γ*S1* mice at early time-points examined and prior to development of overt disease. Mice lacking both SOCS1 and SOCS3 within hematopoietic cells (γ*S1S3*) developed a similar pathology–splenomegaly, multi-organ infiltration by inflammatory cells and accumulation of excess neutrophils and CD8^+^ CD44^hi^ T cells; however the development of disease was dramatically accelerated and in moribund γ*S1S3* mice some aspects of disease were exacerbated in comparison to γ*S1* mice, most notably the extent of neutrophil accumulation in the spleen and blood. As previously observed [17], lack of SOCS3 alone in hematopoietic cells did not result in development of disease. Together, these observations of pathology and disease onset suggest joint control of the immune system by SOCS1 and SOCS3 and show that the absence of SOCS3, while alone well tolerated, significantly accelerates the pathology and inflammatory disease characteristic of SOCS1 deficiency.

Previous studies have established the driving role of T cell deregulation in the development of pathology in mice lacking SOCS1: inflammation is lymphocyte-dependent and un-controlled activation, proliferation and impaired deletion of auto-reactive CD8^+^ T cells underpins the autoimmunity in SOCS1-deficient animals [13]. Consistent with these studies, CD8^+^ lymphocytosis was a prominent characteristic of the inflammatory disease in γ*S1* and γ*S1S3* mice, was present well before disease symptoms manifest and was specific to SOCS1-deficient marrow recipients. Prior to disease, the CD8^+^CD44^hi^ cells in γ*S1* and γ*S1S3* mice did not express typical activation markers such as CD25, CD69, KLRG-1 and PD-1 but did display a number of homing markers including CD49d, CXCR3, ICAM-1 and CD11a. This phenotype was similar to that observed for CD8^+^CD44^hi^ cells in control *S3* and *TAM-* mice; however the numbers of these cells in γ*S1* and γ*S1S3* mice was dramatically elevated. A distinctive characteristic of moribund γ*S1S3* mice, less obvious in their γ*S1* counterparts, was that the majority of CD3^+^CD44^hi^ cells expressed low levels of CD62L, suggestive of particular accumulation of effector memory T cells. As observed in previous models of SOCS1-deficiency [12,13], the accumulation of CD8^+^CD44^hi^ cells was likely contributed to by increased sensitivity to γc cytokines: CD8^+^ cells from γ*S1* and γ*S1S3* mice responded more robustly than control cells to *in vitro* stimulation with IL-2 plus CD3/CD28 or IL-15, with increased up-regulation of CD44 and/or down regulation of CD62L. Moreover, pro-inflammatory cytokines and/or chemokines such as RANTES, MIP1α, IL-3, GM-CSF and TNFα were produced in excessive amounts by IL-2- and/or IL-15-stimulated γ *S1S3* T cells and to a lesser extent also by γ*S1* cells.

Accumulation of neutrophils associated with uncontrolled responses to cytokines such as G-CSF and IL-6 is a defining characteristic of SOCS3-deficient hematopoiesis [15–17]. Accordingly, the progressive neutrophilia observed in γ*S1S3* mice was associated with G-CSF and IL-6 hypersensitivity in isolated neutrophils and macrophages as well as an elevated number of G-CSF-responsive myeloid progenitors in the bone marrow. This hypersensitivity was not observed in γ*S1* cells, nor was it more dramatic in γ*S1S3* cells than in those lacking only SOCS3. Thus, while the neutrophilia was more profound and significantly accelerated in γ*S1S3* mice compared with that in γ*S1* animals, our data provide no evidence to support increased intrinsic sensitivity of SOCS1/SOCS3 double deficient cells over those lacking only SOCS3 and by implication any role for SOCS1 in directly regulating G-CSF and IL-6 signalling pathways, even in the absence of SOCS3. Similarly, while the number of GM-CSF-responsive granulocyte-CFC were increased in γ*S1S3* mice, no evidence for direct regulation of the GM-CSF signalling pathway by SOCS1 and/or SOCS3 was evident; this observation was likely the result of increased levels of GM-CSF in these mice. Finally, G-CSF was only present in elevated concentrations in the serum of moribund γ*S1* mice and not γ*S1S3* animals. Thus, the accelerated onset of neutrophilia and inflammatory disease in γ*S1S3* mice compared to γ*S1* mice appears driven by hyper-responsiveness to normal levels of G-CSF (as a result of SOCS3 deletion), in concert with increased levels of other pro-inflammatory cytokines, while the later onset neutophilia and disease in γ*S1* mice is dependent upon the development of high levels of G-CSF. The precise mechanism by which high concentrations of G-CSF develop in γ*S1* mice is unclear; however it is reasonable to speculate that accumulated production by the high numbers of T cells in these mice is likely to contribute.

Together, our studies establish the importance of joint regulation of the immune system by SOCS1 and SOCS3 in controlling inflammation. The findings of previous studies and the data presented here support a model in which, in γ*S1* mice, the early accumulation of pro-inflammatory CD8^+^ CD44^hi^ T cells, driven at least in part by hypersensitivity to cytokines such as IL-2 and IL-15 is a key initiating step in the subsequent pathology. The expression of homing markers on these T cells may facilitate migration to organs and tissues, and the propensity for production of pro-inflammatory cytokines and chemokines, such as RANTES, capable of further attracting T cells, and MIP1α, GM-CSF and IL-3 for recruiting myeloid cells, as well as development of high circulating G-CSF concentrations, can account for the pathological infiltration of neutrophils, monocytes, eosinophils and lymphocytes at the numerous sites of inflammation including the skin, liver, lung and duodenum that typifies the inflammatory disease that develops in γ*S1* mice. Although the loss of SOCS1 is ultimately unable to be overcome, SOCS3 clearly plays a significant role in delaying the severity of disease. Our data extend the model to suggest that the absence of SOCS3 in the already pro-inflammatory environment established by SOCS1 deficiency, results in hyper-responsiveness of immune cells to cytokines such as G-CSF and IL-6, even in modest amounts, and dramatically accelerates myeloid proliferation and inflammatory infiltration of target tissues.

Maintenance of immune homeostasis involves the complex interplay of multiple immune cells and cytokine actions. SOCS1 and SOCS3 are required together to prevent the normal interplay of these factors, essential to fight infection and for tissue repair, from escalating into uncontrolled activity and rapid inflammation. While our data cannot exclude direct co-regulation by SOCS1 and SOCS3 on specific cytokine pathways, there was no evidence for this in the key signal transduction pathways identified as driving disease in γ*S1S3* mice, such as the γc cytokines and G-CSF. Rather, the data support SOCS1 and SOCS3 working independently at a cytokine and individual cell level to control the proliferation and activation of immune cells but are together essential to ensure regulated coordination of lymphoid and myeloid cell activity in healthy immunity and to prevent excessive activity associated with rapid onset inflammatory disease.

## Supporting Information

S1 Fig Experimental design for generation and analysis of mice lacking both SOCS1 and SOCS3 within the hematopoietic system. (**A**) Generation of mice lacking SOCS1, SOCS3 or both in the hematopoietic system. Experimental mice were highly reconstituted (>80% CD45.2^+^) wild-type recipients of bone marrow from the specific donor genotypes indicated that were subsequently treated with tamoxifen (+) or vehicle (-). These included solely IFNγ-deficient (γ); SOCS1-deficient (γ*S1*); SOCS3-deficient (γ*S3*); and SOCS1, SOCS3 double deficient (γ*S1S3*) as well as controls with hematopoiesis lacking only SOCS3 on a *Ifng*^*+/+*^ background (*S3*) and mice with functionally normal hematopoiesis: (*S3*^*fl*^) and tamoxifen treated (TAM+) or vehicle treated (TAM-) recipients of *Ifng*^*+/+*^*;Socs1*^*+/+*^*;Socs3*^*+/+*^*;ERT2* marrow. The CreERT2 allele was heterozygous in all mice. (**B**) Experimental workflow. Highly reconstituted experimental mice were treated with tamoxifen or vehicle 6 weeks after transplantation. Analysis was performed on separate cohorts at 14 days, 50 days and 180 days following treatment or of individual mice upon signs of disease (moribund). (**C**) Southern blot exemplifying highly efficient Cre-ERT2-dependent recombination of the floxed *Socs3* allele in the hematopoietic organs of tamoxifen (TAM, +) but not vehicle (-) treated mice. WT(+), wild-type allele, fl, floxed allele, Δ, recombined, deleted allele. BM, bone marrow; LN, lymph node.
(TIF)

S2 Fig Altered bone marrow granulocyte and spleen T cell numbers and pathology in mice lacking SOCS1 and/or SOCS3. Representative flow cytometry profiles from analysis of (**A**) neutrophils (Gr-1^+^ CD11b^+^) in the spleen, bone marrow and blood or (**B**) CD8^+^ T cells in the spleens of mice at 50 days following tamoxifen or vehicle treatment, or upon signs of disease (moribund). Absolute numbers of cells are shown in Fig 2; while proportions of CD8^+^ T cells in the spleens of moribund mice were not elevated, absolute numbers were increased due to splenomegaly.
(TIF)

S3 Fig Cellular composition of the bone marrow and spleen in mice lacking SOCS1 and/or SOCS3 in hematopoietic cells. Numbers of myeloid (CD11b^+^ and CD11b^+^ Gr1^+^), B-lymphoid (B220^+^) and T-lymphoid (CD3^+^) cells in bone marrow (**A**) and spleens (**B**) of mice at the indicated times following tamoxifen or vehicle treatment. Means ± SD are shown. * p<0.05 for comparison of γ*S1S3* (moribund) with all genotypes at day 50 (bone marrow: CD11b^+^, CD11b^+^/Gr-1^+^, B220; spleen: CD11b^+^, CD11b^+^/Gr-1^+^), with all genotypes at day 50 excluding γ*S1* (spleen, CD3), with γ*S1S3* (day 14, bone marrow and spleen CD11b^+^, CD11b^+^/Gr-1^+^), and with γ*S1* (moribund, spleen CD11b^+^, CD11b^+^/Gr-1^+^). ** p<0.05 for comparison of γ*S1* (moribund) with all genotypes (bone marrow B220, CD11b^+^, CD11b^+^/Gr-1^+^) at day 180 except *γS1* (non-moribund, day 180), one-way ANOVA with Tukey’s multiple comparisons test, n = 6–17 mice per group. Ratio of donor-derived (CD45.2^+^) B220^+^/CD3^+^ (**C**) cells and CD4^+^/CD8^+^ (**D**) cells in the spleens of mice at the indicated times following tamoxifen or vehicle treatment. Each data point represents an individual mouse with Means ± SD shown. * p<0.05 for comparison of γ*S1S3* (moribund) with all genotypes at day 50 excluding γ*S1*, one-way ANOVA with Tukey’s multiple comparisons test, n = 7–18 mice per group.
(TIF)

S4 Fig Pathology in mice lacking SOCS1 and SOCS3 in hematopoietic cells. Representative photomicrographs of organs from diseased γ*S1S3* mice at low (left panels, ×40: lung, skin, ×100: bone marrow, spleen, duodenum, ×200: liver) and high (right panels, ×600) magnification.
(TIF)

S5 Fig Cytokine signalling in mice lacking SOCS proteins. Representative flow cytometry profiles of PhosFlow measurement of phosphorylated STAT3 (**A**) and STAT5 (**B**) in granulocytes prepared from mice 14d after tamoxifen or vehicle treatment. Cells were stimulated with G-CSF (**A**) or GM-CSF (**B**) for the times indicated.
(TIF)

S6 Fig Interleukin-6 signalling in mice lacking SOCS proteins. Original uncropped western blots of protein lysates from macrophages prepared from mice 14d after tamoxifen or vehicle treatment. The cells were stimulated with IL-6 for the times indicated. Proteins were separated by polyacrylamide gel electrophoresis, transferred to membranes and probed with antibodies to the molecules indicated at the right. Replicate filters were prepared from the same lysates and probed with pSTAT1 and pSTAT3 and subsequently with STAT1 and STAT3 and SOCS3.
(TIF)

S1 Table Peripheral blood cell counts in mice with hematopoiesis lacking SOCS1 and or SOCS3. Mice were bled at the indicated times following tamoxifen or vehicle treatment or upon signs of disease (moribund) for automated blood cell analysis. Means ± SD are shown, n = 10–29 mice per group. * p<0.05 for comparison of γ*S1S3* (moribund) with all other genotypes at day 50, with γ*S1* (moribund) and with γ*S1S3* (day 14), one-way ANOVA with Tukey’s multiple comparisons test.
(DOCX)
