# Supplementary figures and images for: Rapid Inflammation in Mice Lacking Both SOCS1 and SOCS3 in Hematopoietic Cells
Source: PLoS One. 2016 Sep 1;11(9):e0162111. doi: 10.1371/journal.pone.0162111 (PMC5008821; doi:10.1371/journal.pone.0162111)

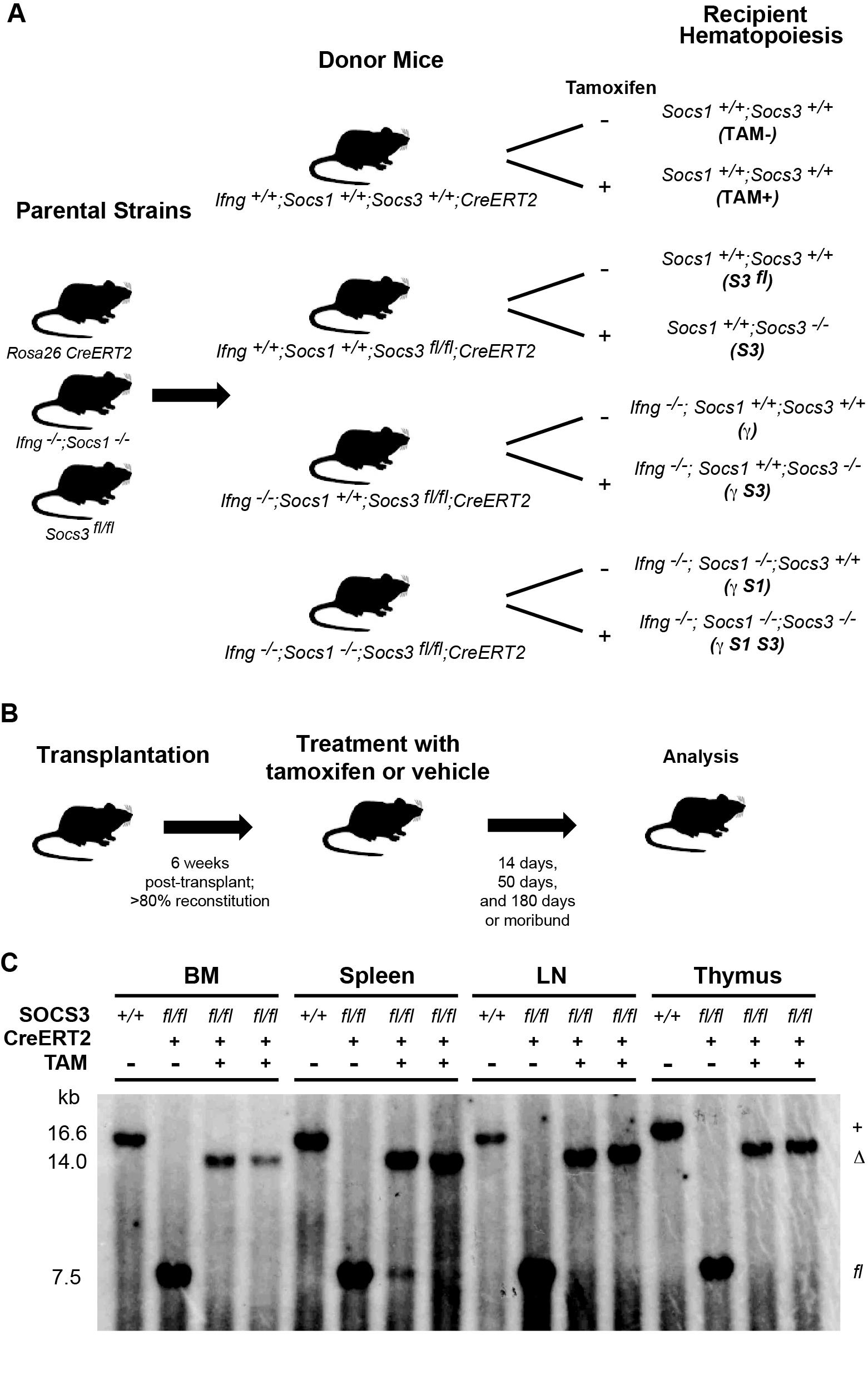

Supplement: S1 Fig — (A) Generation of mice lacking SOCS1, SOCS3 or both in the hematopoietic system. Experimental mice were highly reconstituted (>80% CD45.2+) wild-type recipients of bone marrow from the specific donor genotypes indicated that were subsequently treated with tamoxifen (+) or vehicle (-). These included solely IFNγ-deficient (γ); SOCS1-deficient (γS1); SOCS3-deficient (γS3); and SOCS1, SOCS3 double deficient (γS1S3) as well as controls with hematopoiesis lacking only SOCS3 on a Ifng+/+ background (S3) and mice with functionally normal hematopoiesis: (S3fl) and tamoxifen treated (TAM+) or vehicle treated (TAM-) recipients of Ifng+/+;Socs1+/+;Socs3+/+;ERT2 marrow. The CreERT2 allele was heterozygous in all mice. (B) Experimental workflow. Highly reconstituted experimental mice were treated with tamoxifen or vehicle 6 weeks after transplantation. Analysis was performed on separate cohorts at 14 days, 50 days and 180 days following treatment or of individual mice upon signs of disease (moribund). (C) Southern blot exemplifying highly efficient Cre-ERT2-dependent recombination of the floxed Socs3 allele in the hematopoietic organs of tamoxifen (TAM, +) but not vehicle (-) treated mice. WT(+), wild-type allele, fl, floxed allele, Δ, recombined, deleted allele. BM, bone marrow; LN, lymph node. (TIF) [file pone.0162111.s001.tif]

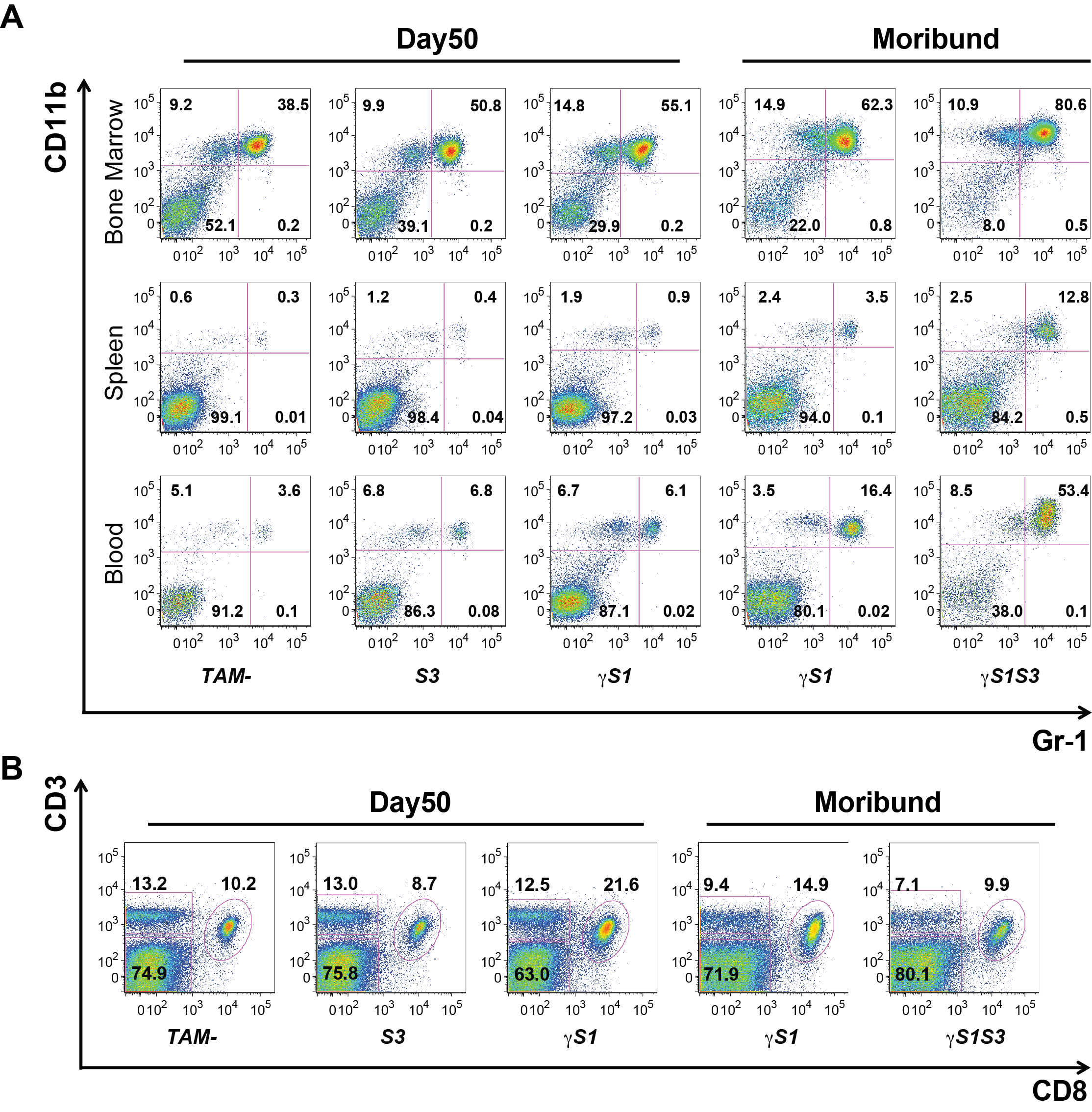

Supplement: S2 Fig — Representative flow cytometry profiles from analysis of (A) neutrophils (Gr-1+ CD11b+) in the spleen, bone marrow and blood or (B) CD8+ T cells in the spleens of mice at 50 days following tamoxifen or vehicle treatment, or upon signs of disease (moribund). Absolute numbers of cells are shown in Fig 2; while proportions of CD8+ T cells in the spleens of moribund mice were not elevated, absolute numbers were increased due to splenomegaly. (TIF) [file pone.0162111.s002.tif]

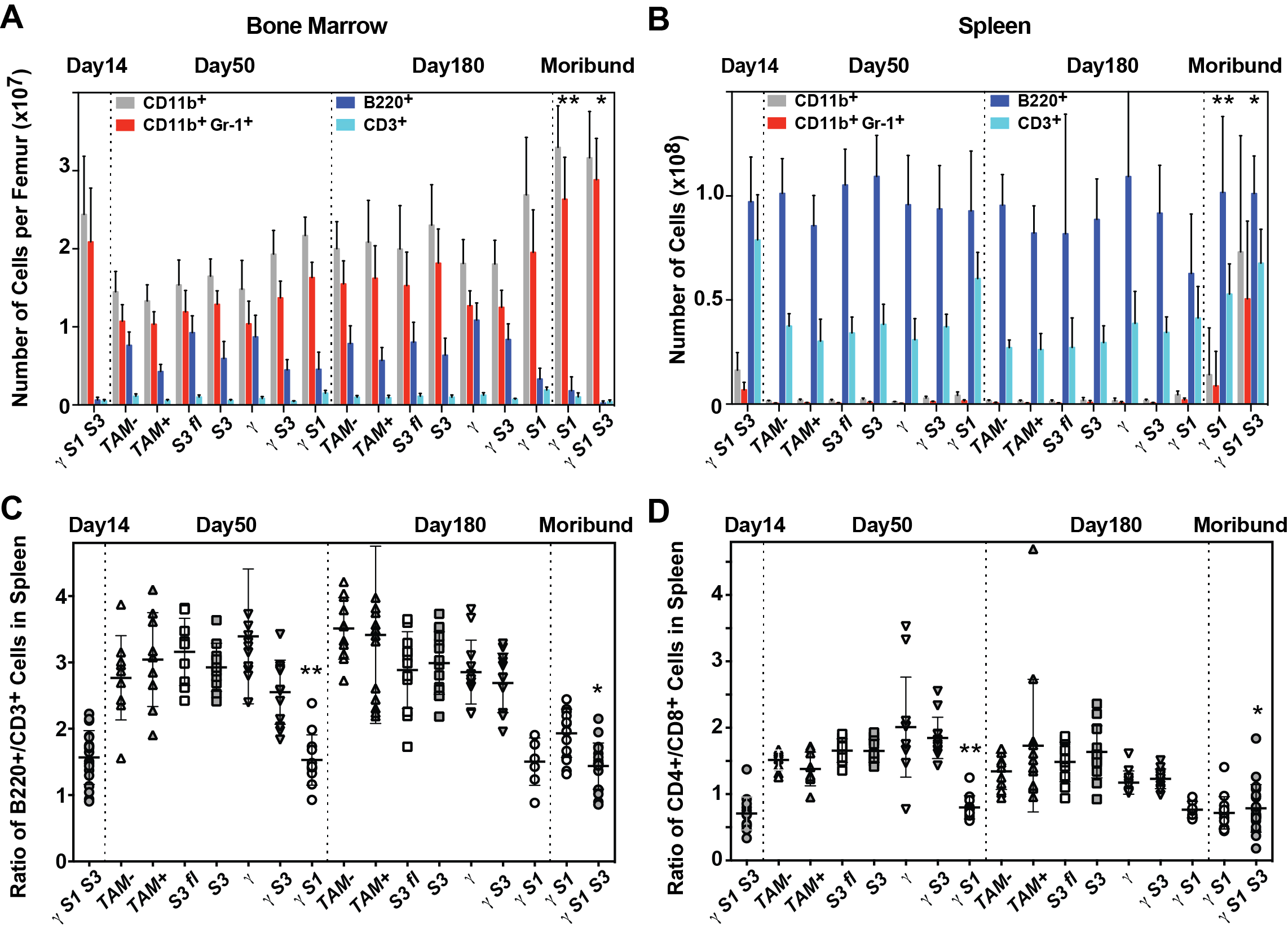

Supplement: S3 Fig — Numbers of myeloid (CD11b+ and CD11b+ Gr1+), B-lymphoid (B220+) and T-lymphoid (CD3+) cells in bone marrow (A) and spleens (B) of mice at the indicated times following tamoxifen or vehicle treatment. Means ± SD are shown. * p<0.05 for comparison of γS1S3 (moribund) with all genotypes at day 50 (bone marrow: CD11b+, CD11b+/Gr-1+, B220; spleen: CD11b+, CD11b+/Gr-1+), with all genotypes at day 50 excluding γS1 (spleen, CD3), with γS1S3 (day 14, bone marrow and spleen CD11b+, CD11b+/Gr-1+), and with γS1 (moribund, spleen CD11b+, CD11b+/Gr-1+). ** p<0.05 for comparison of γS1 (moribund) with all genotypes (bone marrow B220, CD11b+, CD11b+/Gr-1+) at day 180 except γS1 (non-moribund, day 180), one-way ANOVA with Tukey’s multiple comparisons test, n = 6–17 mice per group. Ratio of donor-derived (CD45.2+) B220+/CD3+ (C) cells and CD4+/CD8+ (D) cells in the spleens of mice at the indicated times following tamoxifen or vehicle treatment. Each data point represents an individual mouse with Means ± SD shown. * p<0.05 for comparison of γS1S3 (moribund) with all genotypes at day 50 excluding γS1, one-way ANOVA with Tukey’s multiple comparisons test, n = 7–18 mice per group. (TIF) [file pone.0162111.s003.tif]

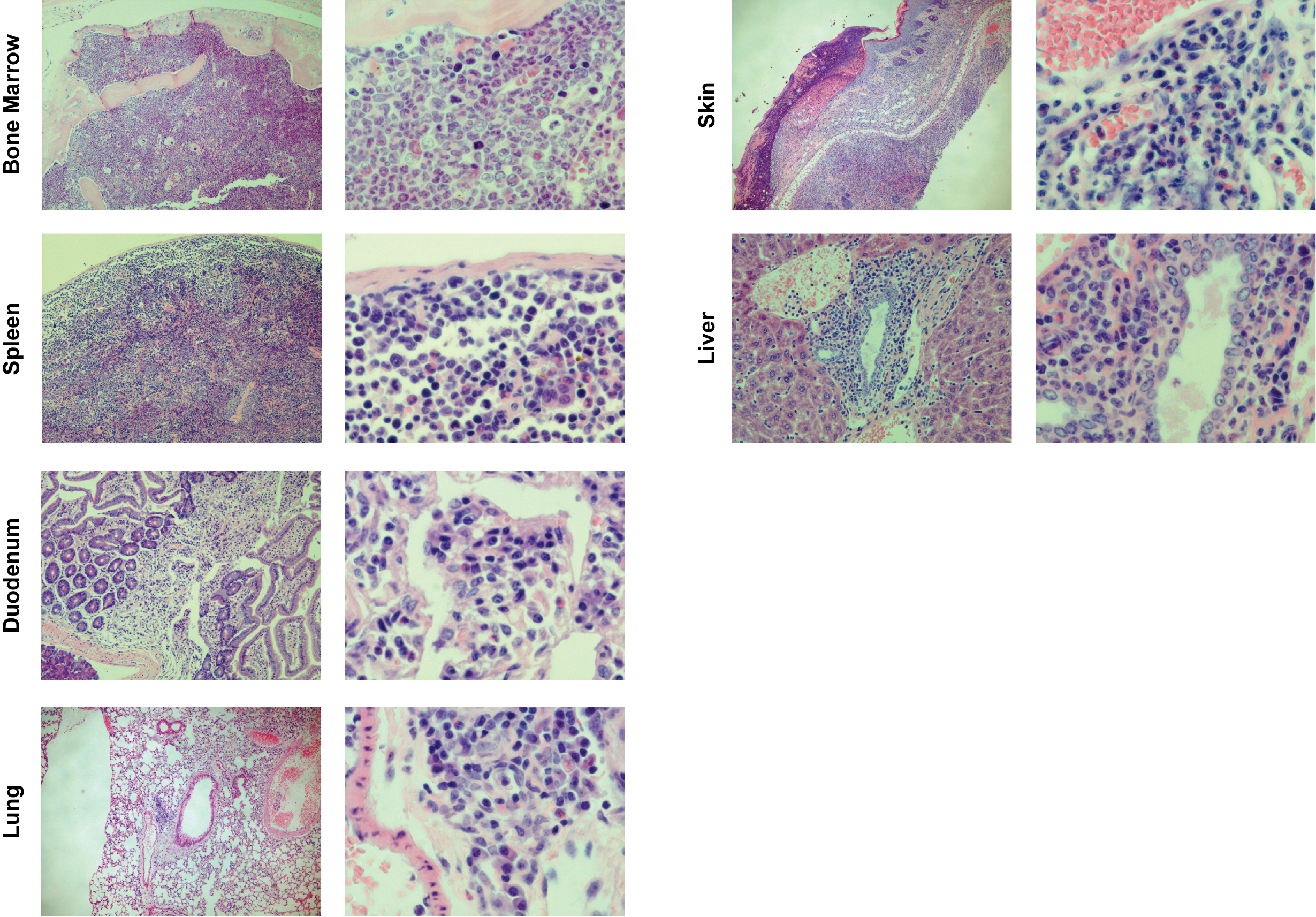

Supplement: S4 Fig — Representative photomicrographs of organs from diseased γS1S3 mice at low (left panels, ×40: lung, skin, ×100: bone marrow, spleen, duodenum, ×200: liver) and high (right panels, ×600) magnification. (TIF) [file pone.0162111.s004.tif]

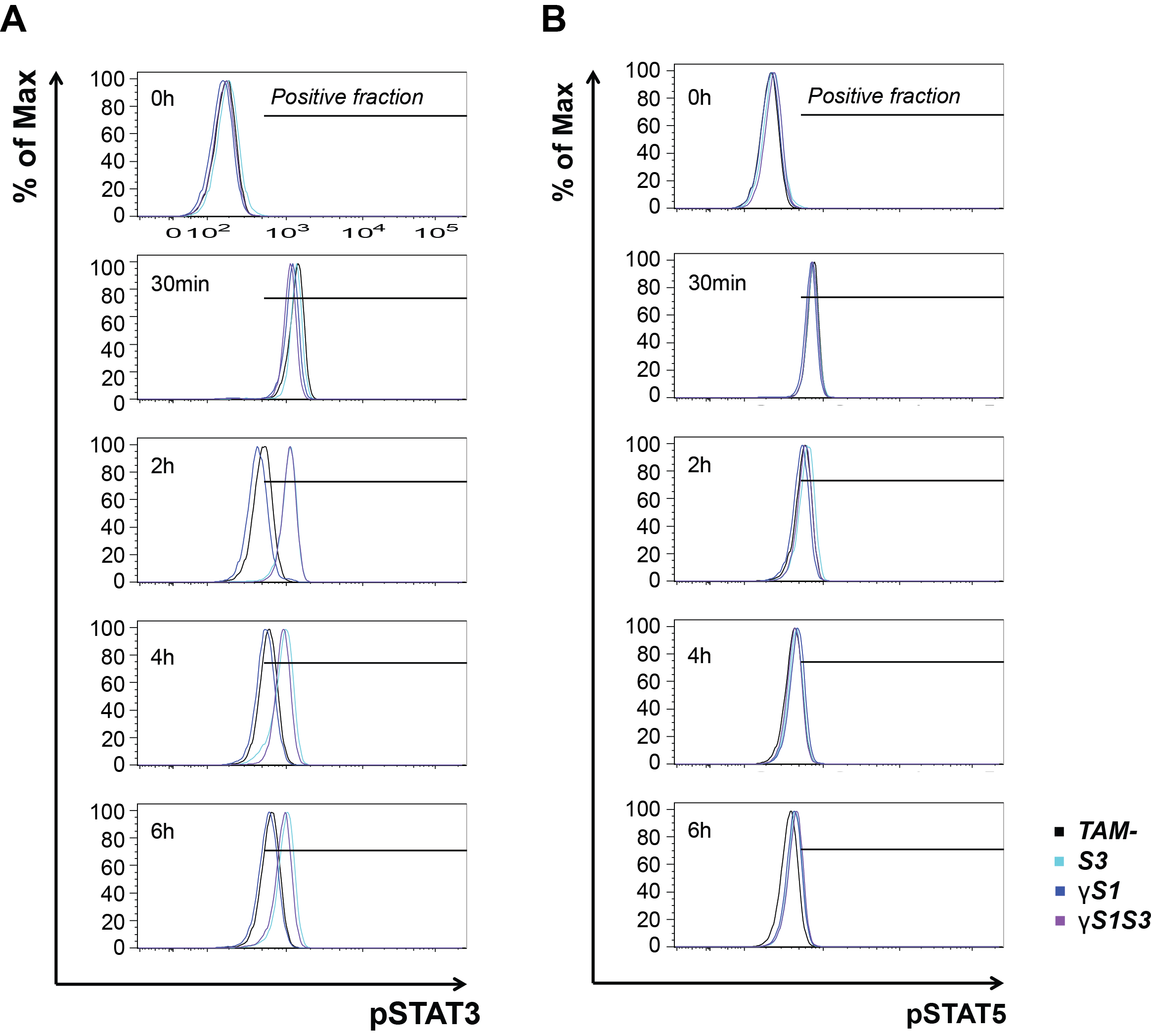

Supplement: S5 Fig — Representative flow cytometry profiles of PhosFlow measurement of phosphorylated STAT3 (A) and STAT5 (B) in granulocytes prepared from mice 14d after tamoxifen or vehicle treatment. Cells were stimulated with G-CSF (A) or GM-CSF (B) for the times indicated. (TIF) [file pone.0162111.s005.tif]

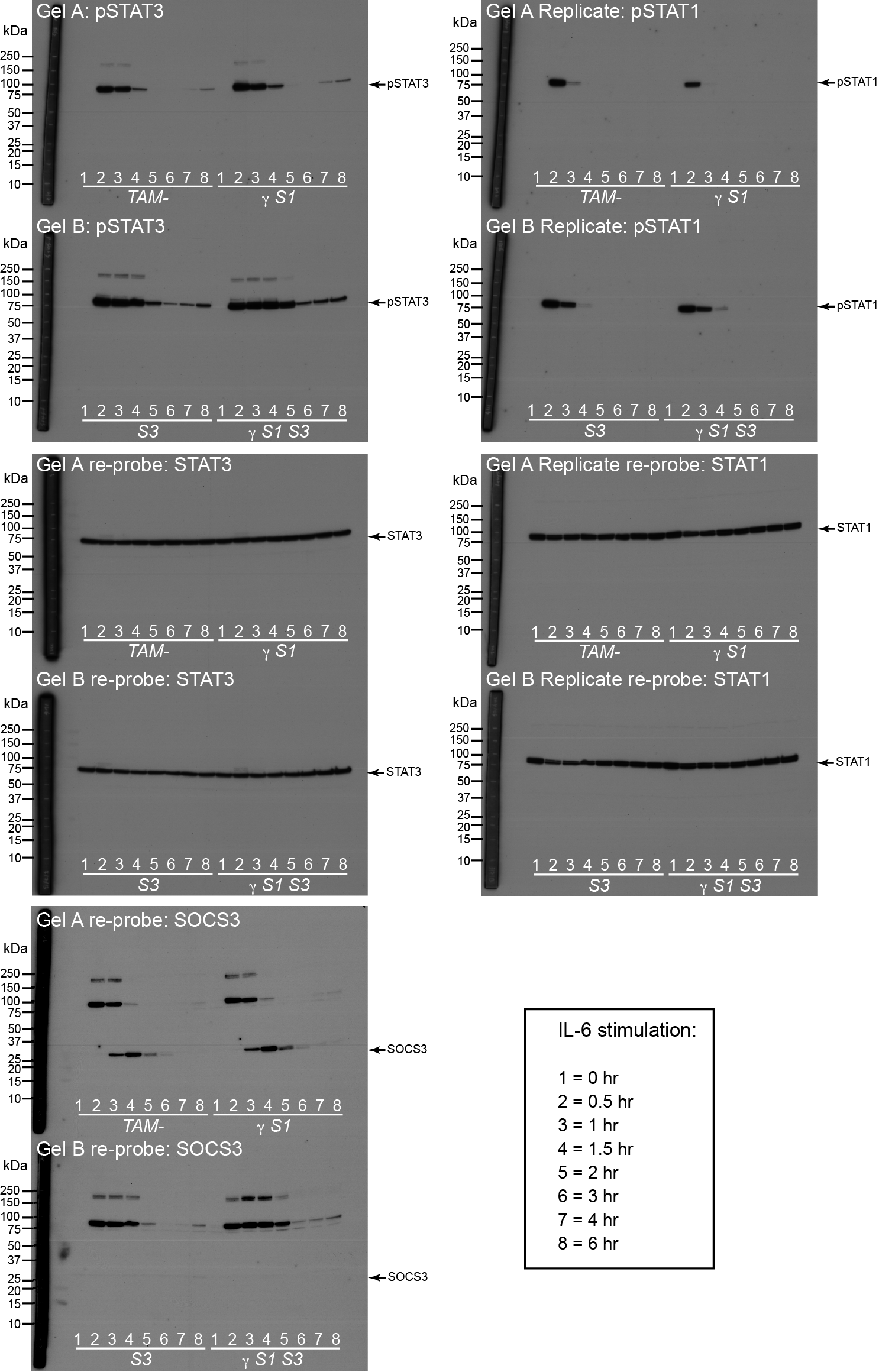

Supplement: S6 Fig — Original uncropped western blots of protein lysates from macrophages prepared from mice 14d after tamoxifen or vehicle treatment. The cells were stimulated with IL-6 for the times indicated. Proteins were separated by polyacrylamide gel electrophoresis, transferred to membranes and probed with antibodies to the molecules indicated at the right. Replicate filters were prepared from the same lysates and probed with pSTAT1 and pSTAT3 and subsequently with STAT1 and STAT3 and SOCS3. (TIF) [file pone.0162111.s006.tif]
