# Supplementary material for: Rapid Inflammation in Mice Lacking Both SOCS1 and SOCS3 in Hematopoietic Cells
Source: PLoS One. 2016 Sep 1;11(9):e0162111. doi: 10.1371/journal.pone.0162111 (PMC5008821; doi:10.1371/journal.pone.0162111)
Supplement: S1 Table — Mice were bled at the indicated times following tamoxifen or vehicle treatment or upon signs of disease (moribund) for automated blood cell analysis. Means ± SD are shown, n = 10–29 mice per group. * p<0.05 for comparison of γS1S3 (moribund) with all other genotypes at day 50, with γS1 (moribund) and with γS1S3 (day 14), one-way ANOVA with Tukey’s multiple comparisons test. (DOCX) [file pone.0162111.s007.docx]

**S1 Table. Peripheral blood cell counts in mice with hematopoiesis lacking SOCS1 and or SOCS3**

|  | **Hb** | **Plt** | **WBC** | **Neu** | **Lym** | **Mono** | **Eos** |
| --- | --- | --- | --- | --- | --- | --- | --- |
|  |  |  |  |  |  |  |  |
| **Day 14** *TAM-* | 15.8 ± 0.4 | 870 ± 138 | 10.7 ± 2.5 | 0.8 ± 0.2 | 9.2 ± 2.3 | 0.11 ± 0.05 | 0.22 ± 0.06 |
| *S3* | 15.2 ± 0.6 | 519 ± 64 | 8.3 ± 3.0 | 0.9 ± 0.3 | 6.8 ± 2.8 | 0.09 ± 0.05 | 0.11 ± 0.04 |
| γ*S1* | 15.0 ± 0.8 | 1036 ± 153 | 13.1 ± 3.0 | 2.1 ± 0.7 | 10.0 ± 2.7 | 0.16 ± 0.06 | 0.40 ± 0.14 |
| γ*S1S3* | 13.1 ± 1.7 | 890 ± 185 | 10.0 ± 3.3 | 2.3 ± 1.3 | 6.5 ± 2.4 | 0.11 ± 0.08 | 0.18 ± 0.11 |
|  |  |  |  |  |  |  |  |
| **Day 50** *TAM-* | 14.2 ± 3.1 | 875 ± 113 | 13.2 ± 2.3 | 0.8 ± 0.1 | 11.7 ± 2.2 | 0.15 ± 0.04 | 0.33 ± 0.31 |
| *TAM+* | 14.8 ± 0.4 | 763 ± 140 | 10.1 ± 2.4 | 0.9 ± 0.3 | 8.5 ± 2.1 | 0.14 ± 0.03 | 0.20 ± 0.06 |
| *S3^fl^* | 15.1 ± 0.4 | 789 ± 115 | 16.3 ± 3.8 | 1.0 ± 0.3 | 14.5 ± 3.4 | 0.18 ± 0.04 | 0.28 ± 0.05 |
| *S3* | 15.1 ± 0.1 | 726 ± 165 | 13.6 ± 2.9 | 1.3 ± 0.3 | 11.6 ± 2.5 | 0.20 ± 0.04 | 0.27 ± 0.08 |
| γ | 15.3 ± 0.4 | 929 ± 92 | 15.9 ± 2.5 | 0.8 ± 0.2 | 14.3 ± 2.4 | 0.16 ± 0.05 | 0.25 ± 0.08 |
| γ*S3* | 15.0 ± 0.3 | 908 ± 113 | 12.5 ± 2.5 | 1.6 ± 0.4 | 10.0 ± 2.4 | 0.22 ± 0.07 | 0.30 ± 0.10 |
| γ*S1* | 14.1 ± 0.7 | 1003 ± 143 | 19.5 ± 5.3 | 3.0 ± 1.2 | 14.9 ± 4.1 | 0.27 ± 0.06 | 0.73 ± 0.22 |
|  |  |  |  |  |  |  |  |
| **Day 180** *TAM-* | 15.1 ± 0.4 | 1160 ± 168 | 11.6 ± 2.3 | 0.9 ± 0.2 | 10.0 ± 2.0 | 0.17 ± 0.14 | 0.21 ± 0.08 |
| *TAM+* | 14.3 ± 0.9 | 1134 ± 225 | 9.0 ± 3.3 | 1.0 ± 0.4 | 7.4 ± 2.8 | 0.13 ± 0.07 | 0.21 ± 0.10 |
| *S3^fl^* | 14.5 ± 1.4 | 1013 ± 152 | 11.1 ± 2.2 | 1.0 ± 0.3 | 9.4 ± 1.8 | 0.13 ± 0.05 | 0.22 ± 0.09 |
| *S3* | 14.5 ± 0.8 | 1103 ± 379 | 11.2 ± 2.1 | 1.4 ± 0.6 | 9.2 ± 1.9 | 0.16 ± 0.04 | 0.22 ± 0.06 |
| γ | 14.2 ± 1.5 | 1064 ± 185 | 15.0 ± 3.2 | 1.1 ± 0.3 | 13.1 ± 2.9 | 0.15 ± 0.04 | 0.38 ± 0.13 |
| γ*S3* | 14.6 ± 1.0 | 1003 ± 143 | 15.6 ± 2.9 | 1.3 ± 0.3 | 13.3 ± 2.6 | 0.25 ± 0.06 | 0.38 ± 0.11 |
| γ*S1* | 13.4 ± 1.0 | 1376 ± 221 | 19.3 ± 6.2 | 4.5 ±1.0 | 13.0 ± 5.1 | 0.23 ± 0.10 | 0.89 ± 0.44 |
|  |  |  |  |  |  |  |  |
| **Moribund** * *γ*S1* | 11.7 ± 2.5 | 1369 ± 312 | 16.1 ± 4.9 | 4.0 ±1.9 | 10.8 ± 2.9 | 0.06 ± 0.03 | 0.43 ± 0.20 |
| γ*S1S3* | 13.3 ± 1.1 | 936 ±321 | 37.2 ± 17.5 * | 22.6 ± 12.8 * | 11.6 ± 4.2 | 0.16 ± 0.11 | 0.77 ± 0.53 |

Mice were bled at the indicated time following tamoxifen or vehicle treatment for automated blood cell analysis. Means ± SD are shown, n=10-29 mice per group. * p<0.05 for comparison of γ*S1S3* (moribund) with all other genotypes at day 50, with γ*S1* (moribund) and with γ*S1S3* (day 14), one-way ANOVA with Tukey’s multiple comparisons test.
